# Multispectral extended depth-of-field fluorescence microscopy with co-designed meta-optics and neural reconstruction

**DOI:** 10.1038/s41377-026-02337-y

**Published:** 2026-05-19

**Authors:** Ipek Anil Atalay Appak, Haobijam Johnson Singh, Sanna Korpela, Teemu O. Ihalainen, Erdem Sahin, Christine Guillemot, Humeyra Caglayan

**Affiliations:** 1 Physics Unit, Faculty of Engineering and Natural Sciences, Tampere University, Tampere, 33720 Finland; 2 INRIA Rennes, Bretagne Atlantique Research Centre, Rennes, 35042 France; 3 Bio Unit, Faculty of Medicine and Health Technology, Tampere University, Tampere, 33520 Finland; 4 Signal Processing Research Centre, Tampere University, Tampere, 33720 Finland; 5 Department of Electrical Engineering, Photonic Integration Group, Eindhoven University of Technology, Eindhoven, 5600 MB The Netherlands

**Keywords:** Wide-field fluorescence microscopy, Metamaterials, Nanophotonics and plasmonics

## Abstract

High numerical aperture fluorescence microscopy provides subcellular resolution, but its depth of field is extremely limited, so thick specimens quickly fall out of focus and typically require axial scanning. Multispectral imaging further compounds this problem because chromatic aberrations shift the best focus plane and distort registration across emission channels, especially in thick samples. In this work, we present MANTIS (Multispectral All-Depth meta-opTic Imaging System), a co-designed computational microscopy imaging system that achieves extended depth-of-field from a single acquisition without axial scanning. MANTIS combines a learned meta-optic with a physics-guided neural network trained end-to-end to reconstruct sharp multispectral images from depth, and wavelength-dependent blurred sensor measurements. We experimentally demonstrate a 50 *μ*m extended depth of field at NA 1.1, corresponding to an 82-fold increase over a conventional wide-field microscope. In addition, simulations show that MANTIS can target different depth-of-field ranges, with the expected trade-off that larger depth-of-field ranges come at a cost in reconstruction fidelity. We validate the approach on biologically relevant fluorescence specimens, including 50 *μ*m thick three-dimensional cultured MDCK II spheroids, and show that, compared with conventional wide-field fluorescence microscopy, reconstructions maintain contrast and lateral detail across depth, with reduced defocus blur and consistent performance across spectral channels.

## Introduction

Fluorescence microscopy is a cornerstone imaging technique in biological and biomedical research. It works by labeling specific molecules in a sample with fluorescent dyes that emit light upon excitation at a specific wavelength. This enables precise, high-contrast visualization of objects such as individual proteins, organelles, and entire cells. To achieve detailed images at the sub-cellular levels, fluorescence microscopes often use high numerical aperture (NA) objectives. A high NA lens can collect more light and resolve finer details. However, this increase in resolution comes with a fundamental limitation: a significantly reduced depth-of-field (DoF), the axial range over which objects appear sharp in an image. As NA increases, DoF shrinks rapidly, often to just a few microns or less^1^. This means that only a thin slice of a 3D biological sample is sharply in focus at any given time, while structures closer to or further away from the in-focus region appear blurred. This shallow DoF is particularly problematic for thick or heterogeneous samples, such as tissue sections, organoids, or even single cells with complex internal structures. In live-cell imaging, the issue is compounded by the fact that the sample can be dynamic and fast-moving, requiring rapid imaging across multiple planes.

Traditionally, microscopes mitigate shallow DoF by axial scanning, acquiring a z-stack by stepping the specimen or the objective along the optical axis. The slices are then fused to produce an all-in-focus image. Confocal microscopy provides optical sectioning by rejecting out-of-focus light with a pinhole. Still, it relies on horizontal and axial scanning to build a volume or to synthesize extended-depth-of-field (EDoF) views. Imaging by scanning increases acquisition time and is prone to motion artifacts in live specimens. Repeated illumination further increases photobleaching and phototoxicity, which limits throughput and temporal resolution. A range of advanced optical techniques has been developed to reduce reliance on mechanical z-scanning, most notably light-sheet microscopy, remote focusing, and spatial or spectral multiplexing. In light-sheet microscopy^2,3^, illumination and detection are decoupled so that only a thin slice of the sample is excited, minimizing out-of-focus background. Remote focusing^4–6^ shifts the focal plane by tuning optical power rather than physically moving components. Spatial and spectral multiplexing^7–9^ simultaneously acquire multiple planes or channels. Despite their strengths, these methods often require elaborate hardware with additional lenses, beam splitters, or custom illumination paths, increasing both cost and system complexity. Many implementations still rely on sequential acquisition across depths or wavelengths, preventing full-volume capture at the camera’s native frame rate. Two-photon microscopy offers deeper penetration but relies on high-peak-intensity pulses, which increase photobleaching and phototoxicity^10,11^, limiting its suitability for live-cell experiments.

Multispectral fluorescence imaging adds an additional challenge on top of the EDoF constraint. The microscope point spread function (PSF) depends on NA, wavelength, and defocus, so any residual chromatic aberration causes the best-focus plane and PSF width to vary slightly between channels. In thick specimens this leads to depth-dependent misregistration: at a given plane some channels are sharp while others are blurred, producing color fringes and cross-channel offsets that complicate co-localization and quantitative analysis across the axial range. A practical system should therefore maintain nearly uniform resolution and accurate cross-channel registration over wavelength and depth from a single exposure per field of view (FoV), without resorting to axial stacking. Although modern high-NA objectives strongly suppress chromatic aberrations, residual wavelength dependence of the PSF and dispersive elements elsewhere in the optical path can still introduce non-negligible chromatic shifts in thick, multi-color samples.

Computational imaging offers a powerful route to multispectral EDoF by jointly designing the optics and the reconstruction algorithm. In wavefront-coding approaches, the PSF is deliberately engineered so that sensor measurements remain informative across a large depth range and can be computationally restored^12–18^. Although effective, most implementations to date rely on diffractive optical elements (DOEs), whose inherent dispersion complicates chromatic correction. This makes it challenging to manage wavelength-dependent aberrations at the physical layer, particularly when multiple spectral channels must be captured simultaneously with high precision.

Meta-optics offers a path beyond these limitations. By providing subwavelength control over phase, dispersion, and polarization within an ultrathin platform, meta-optics enable optical functions far exceeding those of conventional DOEs^19,20^. Leveraging this flexibility, several EDoF metalens architectures combine engineered phase profiles with computational reconstruction to achieve broadband, depth-invariant imaging. Examples include rotationally symmetric EDoF metalenses paired with deconvolution, fast varifocal meta-optics, and inverse-designed lenses that generate uniform focal spots or lens-like PSFs across extended depths^21–25^. These studies demonstrate that meta-optics with post-processing can handle RGB or broadband scenes under defocus. However, the reported designs use moderate NA and are typically evaluated on macroscopic, planar targets, so the EDoF corresponds to relatively mild blur. Consequently, these approaches do not yet enable high NA volumetric multispectral imaging of thick biological specimens in a single shot at high spatial resolution. Parallel advances in achromatic metalens arrays have demonstrated snapshot full-color light-field and integral imaging, offering broadband, multi-view capture within a single exposure^26–28^. However, unlike single-aperture EDoF designs, array-based approaches divide the finite pixel and photon budget across multiple views, inherently reducing per-view resolution and signal-to-noise ratio (SNR).

These developments are particularly relevant in fluorescence microscopy, where spatial resolution, spectral fidelity, and DoF are tightly coupled. Thick, multi-label biological samples demand accurate color registration across depth, while high NA amplify defocus blur differently across different wavelength channels. As a result, existing EDoF meta-optic systems, though promising, still fall short for practical volumetric fluorescence imaging, often requiring axial stacks, multi-plane acquisition, or reduced NA to maintain image quality. The convergence of meta-optics and computational imaging now opens the door to overcoming these limitations. By jointly tailoring the physical wavefront transformation and the reconstruction algorithm, it becomes possible to encode depth and wavelength-dependent information in a single measurement and decode it computationally without sacrificing spatial resolution.

Here we realize this concept in MANTIS, the Multispectral All Depth meta-opTic Imaging System, a co-designed computational microscopy imaging system that addresses both EDoF and chromatic aberration and enables high-resolution multispectral three-dimensional fluorescence imaging from a single shot. MANTIS combines a learned meta-optic with a deblurring convolutional neural network (D-CNN) that is trained together within a physics-based differentiable forward model with a stable multi-term loss, allowing optimization of the full imaging pipeline without axial scanning, color corrected relay optics, or per channel calibration. This co-optimization yields a compact and high-speed system that performs volumetric multispectral reconstruction from a single exposure. In experiments, three-dimensional fluorescence images collected with MANTIS closely track the physics-based simulations, supporting the robustness of the learned design. Together, these results establish MANTIS as a multispectral EDoF imaging approach that delivers high resolution, cross-channel registered volumetric reconstructions in a single acquisition without mechanical scanning or additional corrective optics.

## Results

**Fig. 1:**
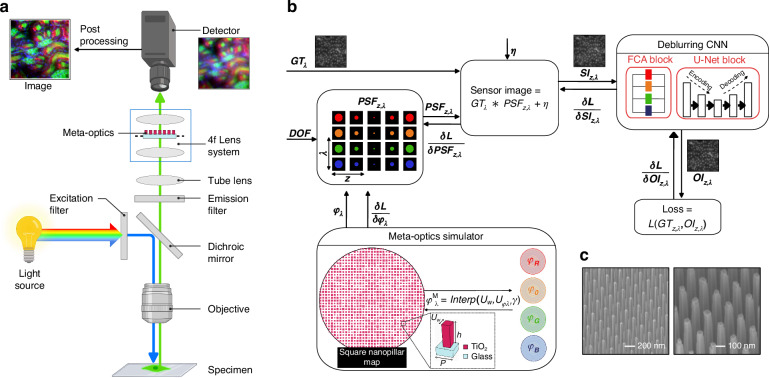
**MANTIS: multispectral all-depth meta-optic imaging system. a** Schematic of the proposed MANTIS system, a 4*f* computational EDoF fluorescence microscopy with a learned meta-optic at the Fourier plane. Fluorescence from a 3D sample is relayed through excitation and emission filters, a dichroic mirror, a tube lens, and an objective lens. A 4*f* system then directs the light through the meta-optic, which modulates the wavefront before a neural network processes it. **b** Differentiable modeling pipeline. A spatially varying width map of square TiO_2_ nanopillars, defined by edge widths *γ*, is used to compute wavelength-dependent phase delays ${\phi }_{\lambda }^{M}$ via a differentiable spectral lookup function ${\phi }_{\lambda }^{M}={\rm{Interp}}({U}_{w},{U}_{{\phi }_{\lambda }},\gamma )$. This phase profile is then used to simulate the PSF_*z*,*λ*_ using a wave-optics model. The ground truth object *GT*_*λ*_ is convolved with these PSFs to form the sensor image *SI*_*z*,*λ*_ = *GT*_λ_*PSF_*z*,*λ*_ + *η*. A convolutional neural network (CNN) reconstructs the object as *OI*_*z*,*λ*_. The entire system is trained end-to-end by minimizing the reconstruction loss ${\mathcal{L}}(G{T}_{z,\lambda },O{I}_{z,\lambda })$. Colors represent different wavelengths *λ* ∈ [433, 681] nm, while the *z*-axis encodes depth variation. **c** Scanning electron microscope (SEM) images of the fabricated meta-optic. Scale bars, 200 nm and 100 nm, respectively

To realize MANTIS, we developed an integrated optical and computational system that jointly addresses chromatic aberration and defocus blur. The overall architecture and learning pipeline are illustrated in Fig. 1.

The optical setup (Fig. 1a) is based on a modified wide-field fluorescence microscope. To access the native Fourier plane within the objective, a secondary 4f relay system is introduced to reimage it externally. An optimized meta-optic, implemented as a TiO_2_ nanopillar array, is positioned at the reimaged Fourier plane to modulate the wavefront before image formation. The captured sensor images are subsequently processed by a trained D-CNN to reconstruct high-resolution sample images across multiple depths and wavelengths.

Figure 1b illustrates the differentiable end-to-end learning framework used to optimize the entire imaging system. During training, high-resolution image patches, annotated with their corresponding depth and spectral channels, are propagated through a physics-based forward model that emulates image formation at the sensor plane. The simulated sensor outputs are then processed by the D-CNN, which is trained to reconstruct the original object. The meta-optic phase profile and network parameters are jointly optimized via backpropagation, enabling co-adaptation of optical encoding and computational decoding toward optimal reconstruction fidelity. After training convergence, the optimized meta-optic is fabricated and integrated into the fluorescence microscope to enable single-shot multispectral EDoF imaging. Further implementation details are provided in the following sections.

### Simulations

Our framework, MANTIS, is developed to address the inherent trade-off between the DoF and spatial resolution in multispectral fluorescence imaging. This relationship is described by

1 $${\rm{DOF}}\propto \frac{\lambda }{{{\rm{NA}}}^{2}}\propto \frac{{{\rm{Resolution}}}^{2}}{\lambda }$$

indicating that achieving a large DOF becomes more challenging with high NA objectives and shorter wavelengths. We use a wide-field fluorescence microscope with a 1.1 NA objective, making the problem particularly demanding.

In simulations, all optical parameters are fixed and identical across experiments (see Section 1 1 in Supplementary). The only configuration-dependent change is the Fourier-plane sampling grid Δ*s*, which is fixed by two criteria: (i) the space-bandwidth product (SBP) required by the passed object-space bandwidth, (ii) and a phase-sampling bound set by the worst defocus at the shortest wavelength. For incoherent imaging, the required space-bandwidth product is given by:

2 $$\begin{array}{rcl}SBP(\lambda ) & \approx & \mathop{\sum }\limits_{\lambda }\,\left[(2{L}_{x}{f}_{{\rm{pass}}}(\lambda ))(2{L}_{y}{f}_{{\rm{pass}}}(\lambda ))\right],\\ {f}_{pass}(\lambda ) & = & min\{{f}_{c}(\lambda ),{f}_{Nyq,obj}\},\,{L}_{x,y}=({N}_{x,y}p)/M\end{array}$$

where *f*_*c*_ = 2*NA*/(*λ*) is the incoherent cutoff spatial frequency, *f*_*Nyq*,*obj*_ = 1/(2*p*/*M*) is the camera Nyquist frequency mapped to the object space, *L*_*x*,*y*_ is the object-space FoV, *N*_*x*,*y*_ the sensor pixel counts, *p* is the sensor pixel size, and *M* is the magnification. For our system, *SBP*(*λ*) is 4.2M, 3.3M, 2.6M, 2.05M at {433, 521, 601, 681} nm, totaling *SBP*_*total*_ = 12.3M.

Furthermore, to prevent PSF aliasing, we impose an upper bound on the Fourier sampling grid. The critical Fourier sampling grid, Δ*s*_crit_, is bounded by the maximum phase slope at the pupil edge, which is determined by the maximum defocus at the shortest wavelength^14^:

3 $$\Delta {s}_{{\rm{crit}}}=\frac{\pi (D/2)}{8\,{\psi }_{\max }({\lambda }_{\min })},\,{\psi }_{z}=\frac{\pi }{\lambda }\left(\frac{1}{z}-\frac{1}{{z}_{0}}\right){(D/2)}^{2}$$

where *ψ*_*z*_ is the defocus coefficient, *D* is the entrance pupil diameter, *z*0 is the nominal focus and ${\psi }_{max}=max(| {\psi }_{{z}^{-}}| ,| {\psi }_{{z}^{+}}| )$ over the target DOF. We then oversample this critical grid value so that the resulting Fourier sampling pitch supports the required total SBP across all wavelengths and reduces phase error and numerical aliasing:

4 $$\Delta s=\frac{1}{\alpha }\,\Delta {s}_{{\rm{crit}}},\,\alpha =\frac{SB{P}_{{\rm{total}}}}{SBP({\lambda }_{\min })}$$

With *α* = 4, the resulting Δ*s* oversamples the critical grid and provides a more than threefold margin in space bandwidth relative to the SBP requirement at the shortest wavelength, which reduces numerical error and improves PSF accuracy and reconstruction quality across wavelengths. Table 1 in Supplementary, reports the effect of the oversampling factor *α* on final performance.

Using these constraints, we trained configurations targeting EDoF ranges of 25, 50, 75 μm. Table 1 summarizes the Δ*s* and *ψ*_*max*_ for each target training depth. To balance accuracy and computational efficiency, we set *K* = 5 training depths for the 25 μm and 50 μm targets, and *K* = 7 for 75 μm. Increasing *K* beyond these values did not improve the performance. With the multispectral meta-optic, PSFs vary smoothly with depth and differ across wavelengths, so a few well-spaced planes already cover the intermediate depths. Denser sampling adds redundant targets, and small wavelength-dependent differences increase the gradient noise without improving accuracy.

**Table 1:** Quantitative comparison of trained configurations targeting different EDoF ranges

Target DoF	*ψ*_*max*_	Δ*s* (*μ*m)	PSNR (dB)	SSIM
25 μm	110.20	2.25	24.99	0.78
50 μm	221.32	1.125	23.53	0.70
75 μm	333.38	0.675	22.51	0.68

PSNR and SSIM values are averaged over depth and spectral channels

For all configurations, we implemented the network in PyTorch with two Adam optimizers: one for the optical parameters (learning rate 10^−2^) and one for the D-CNN (learning rate 10^−4^, weight decay 10^−3^). Training and evaluation were performed on the same mixed dataset described in Section 4.3, using an NVIDIA Tesla V100 SXM2 with 32 GB memory. On average, each model required 44 hours and 36 minutes of training time. After training, during inference the D-CNN processes a four-channel 512 × 512 patch in 7.53 ms on average, corresponding to 132.7 patches per second on our hardware.

For quantitative evaluation, we assessed performance on 47 multispectral test images from our mixed dataset, across 41 uniformly spaced depth planes in diopters to cover the full axial range. Each four-channel image was assigned to a discrete depth, and metrics were aggregated over all depths and channels. Table 1 summarizes results for the target EDoF ranges using peak signal-to-noise ratio (PSNR) and structural similarity index (SSIM). As expected, there is a gradual decrease in PSNR and SSIM as the targeted DoF increases from 25 to 75 μm, reflecting the increased difficulty of reconstructing scenes under stronger defocus.

**Fig. 2:**
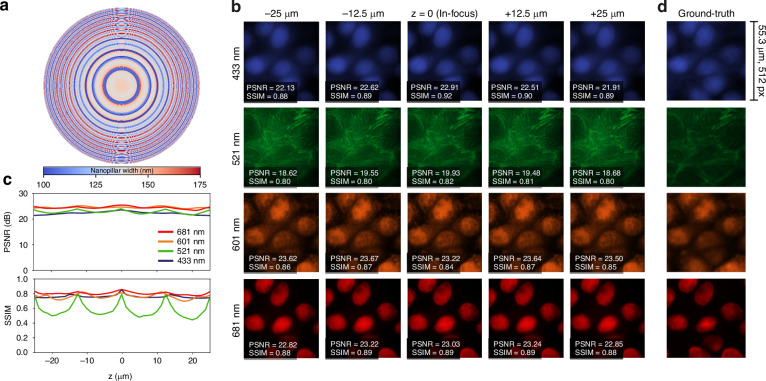
**Simulation results of multispectral 50** **μm EDoF learning. a** End-to-end-learned multispectral meta-optic layout shown as a nanopillar-width map. **b** Simulated reconstructions of a 4-channel test patch at five axial positions spanning the target DoF. Rows correspond to emission wavelengths 433, 521, 601, and 681 nm. PSNR and SSIM are annotated for each tile. **c** PSNR and SSIM as a function of depth for the multispectral design across 41 axial planes within the DoF. **d** Ground-truth 4-channel test patch used for the simulations in (**b**)

For a target DoF of 50 μm, Fig. 2a displays the end-to-end-optimized meta-optic rendered as a nanopillar-width map. Representative reconstructions across wavelengths and axial positions are shown in Fig. 2b, where the ground-truth *GT*_*λ*_ is identical for all axial positions and is shown in Fig. 2d. Figure 2c reports the corresponding PSNR and SSIM versus depth for each spectral channel. The PSNR remains largely invariant with depth and exhibits minimal inter-channel variation, demonstrating stable pixel-level fidelity across the extended focal range. SSIM follows a similar trend for the 433, 601, and 681 nm channels; however, the 521 nm channel shows a decline outside the trained depth interval. This behavior likely reflects channel specific structure in the MDCK dataset, which consists of four channel fluorescence images of Madin Darby canine kidney epithelial cells labeled with fluorophores emitting at 433, 521, 601, and 681 nm and acquired on a Nikon ECLIPSE Ti2 microscope, where the 521 nm channel emphasizes finer, lower contrast features that are intrinsically more challenging to reconstruct (see “Methods” and “Sample preparation” for details).

For comparison, earlier EDoF studies typically use small NA objectives and a restricted depth range, which leads to much smaller values of the defocus coefficient ${\psi }_{\max }$, defined in Eq. (3). In contrast, our configuration uses a high NA and substantially larger target depth ranges, resulting in significantly higher ${\psi }_{\max }$ values. For reference, reported values are ${\psi }_{\max }\approx 81.99$ for E2E-BPF^29^, ${\psi }_{\max }\approx 24.63$ for DeepDOF-SE^30^, and ${\psi }_{\max }\approx 24.59$ for Zhang et al.^31^. In our study, ${\psi }_{\max }$ ranges from 110 to 333 for the 25 μm, 50 μm, and 75 μm target DoFs, respectively. This indicates that our optimization operates under defocus blur conditions at least three times stronger than those considered in prior works. Moreover, while several of the compared designs are limited to a single wavelength, our framework is jointly trained and evaluated in a multispectral setting, further increasing the complexity of the optimization.

In addition to the multispectral design, we trained a single-wavelength meta-optic with a single-channel reconstruction network targeted at the 601 nm emission band. This model follows the UNet configuration from our recent work^32^ and omits the spectral Fourier domain attention block, since only a single channel is present and no cross-channel reweighting is needed. As detailed in Section 2 in the Supplementary, the single wavelength model achieves the highest PSNR and SSIM at its design wavelength of 601 m, while performance at the other emission bands is lower. In contrast, the multispectral design sacrifices a small amount of peak performance at 601 nm and yields PSNR and SSIM that remain more uniform across wavelengths and depth, which is advantageous for quantitative multi-label fluorescence imaging. Together, these comparisons indicate that MANTIS learns a jointly optimized multispectral EDoF response with stable reconstruction quality across both wavelength and depth.

### Experimental results

To validate MANTIS, we fabricated the multispectral meta-optic designed for a 50 μm target EDoF and integrated it into the 4f relay described above. As reference configurations, we use a conventional wide-field microscope and a single wavelength meta-optic optimized at 601 nm. We evaluate these configurations via PSF measurements and reconstructions of biological samples, as detailed below. Unless stated otherwise, wide-field and meta-optic images are acquired in parallel using two arms of the same microscope, providing a frame matched baseline for each FoV (see Supplementary Fig. 1 and Supplementary Section 1).

#### PSF characterization

**Fig. 3:**
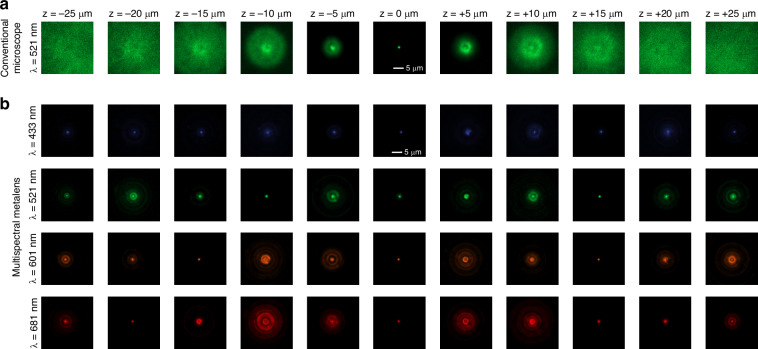
**Experimental PSFs across 50** **μm target DOF.** PSFs measured from 1 μm fluorescent beads for (**a**) a conventional microscope, **b** our multispectral meta-optic. For each configuration, PSFs are presented at 11 discrete axial positions spanning a 50 μm target DOF (−25 μm to +25 μm relative to best focus) and are shown with identical intensity scaling. Plots report the lateral FWHM versus depth, highlighting the stability of our designs. Scale bars, 5 μm

We experimentally characterized the fabricated meta-optic and the reference wide-field microscope by measuring PSFs from 1 μm fluorescent beads embedded in a polyacrylamide hydrogel under LED excitation at each emission wavelength. The sample was axially scanned over a 50 μm range with 500 nm step size using a piezo stage, and fluorescence images were captured at each axial position to obtain a three-dimensional PSF stack. Experimental PSFs for the conventional microscope and for the multispectral meta-optic are shown in Fig. 3. Compared with the wide-field microscope, its PSFs vary much less with defocus and with wavelength, indicating reduced axial sensitivity and improved lateral invariance across the scan. For comparison, simulated PSFs computed under the same conditions are shown in Supplementary Fig. 6 and agree well with the measurements, supporting that the meta-optic encodes the intended spectral phase profile. For completeness, we applied the same protocol to the learned single wavelength meta-optic and report the corresponding measured PSFs at each emission band in Supplementary Fig. 3.

To characterize PSF width and its variation with depth for the multispectral meta-optic, we fitted a two-dimensional Gaussian to the measured PSFs at each wavelength and axial plane and extracted the lateral full width at half maximum (FWHM). We displayed these fits as horizontal and vertical line profiles with the Gaussian curves overlaid, and the corresponding FWHM values indicated on each panel. Plots for all four emission bands and all depth planes are provided in Supplementary Section 3. The resulting FWHM curves are consistent with achromatic EDoF behavior of the learned multispectral meta-optic and show only modest broadening over depth and wavelength.

We note that metasurface fabrication can introduce deviations from the designed nanopillar geometry, which affect the realized wavelength-dependent phase profile and can shift the measured PSF relative to the nominal design. This introduces a model mismatch that may reduce reconstruction fidelity when deviations are sufficiently large. In our framework, robustness can be improved by calibrating the forward model using experimentally measured PSFs of the fabricated meta-optic and, if needed, fine tuning the reconstruction network on calibration data. In the present experiments, this additional fine tuning was not required, since the measured PSFs closely followed the expected depth and spectral trends. A quantitative analysis of fabrication induced geometry perturbations for learned meta-optics and their impact on imaging performance is provided in our prior work^32^.

#### Reconstruction of biological samples

We evaluated MANTIS on three biological specimen types: MDCK epithelial monolayers, three-dimensional cultured MDCK II spheroids (≈50 μm thick), and a mouse kidney cryosection (FluoCells Prepared Slide 3, F24630, 16 μm thick). Detailed preparation and staining protocols are provided in “Methods”, Section “Sample preparation”).

We first imaged MDCK epithelial monolayers stained with the same four fluorescence markers used in the dataset, namely DAPI for nuclei, Atto Fluor 640 for F-actin, Alexa Fluor 568 for vimentin, and Alexa Fluor 488 for lamin A, thereby capturing both nuclear and cytoskeletal structures. For each FoV, we acquired paired measurements on a standard wide-field microscope and on the microscope incorporating the learned multispectral meta-optic. In both cases, the sample was translated axially through the target DoF and imaged at five discrete planes. Only the meta-optic measurements were processed through the end-to-end trained D-CNN, while the wide-field images are shown as acquired to provide a baseline reference.

**Fig. 4:**
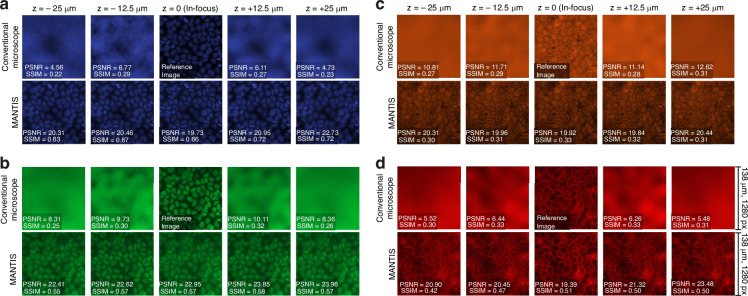
**MDCK cell imaging across the target DOF.** The sample is translated axially across the target DOF, and we compare: (i) conventional microscope, (ii) MANTIS. Four emission channels: **a** 433 nm, **b** 521 nm, **c** 601 nm, and **d** 681 nm. For each channel, the sample is translated through the target DOF and five axial planes. Across all wavelengths, our method consistently resolves cellular structure throughout the DOF, whereas the conventional microscope exhibits strong defocus blur at off-focus positions

Figure 4 compares MDCK images across depth and wavelength. In the wide-field images, cellular morphology rapidly deteriorates with defocus, and blur dominates the contrast. Using our approach, fine cellular features remain discernible throughout the targeted DoF. For quantitative assessment, we took the in-focus wide-field image as the reference when computing PSNR and SSIM as functions of depth. Within each spectral channel, both metrics exhibit only modest variation with axial position. These experimental trends align with our simulated performance across depth and wavelength and are consistent with the low chromatic aberration of the meta-optic. During inference, on an NVIDIA Tesla V100 SXM2 with 32 GB memory, D-CNN processed 1280 × 1280 pixel patches in 46.97 ms on average (21.3 patches per second).

Using these experimental results, we further assessed the role of co-design by quantifying the contributions of the meta-optic and the D-CNN in Supplementary Section 4. We compared three configurations: the full system with both meta-optic and D-CNN, a system with the meta-optic removed, and a system with the D-CNN removed, all based on the same D-CNN architecture. These ablation experiments show that neither component on its own matches the performance of MANTIS in our experiments, highlighting the importance of joint optimization of optics and reconstruction.

For completeness, we repeated the experiment with the single wavelength learned meta-optic optimized at 601 nm and reconstructed those measurements with the 1-channel D-CNN. The PSF measurements were well-formed across wavelengths, yet the reconstructions show strong chromatic dependence. Image quality is strongly wavelength dependent, with PSNR and SSIM varying substantially across channels. Results are provided in Section 2 in the Supplementary.

**Fig. 5:**
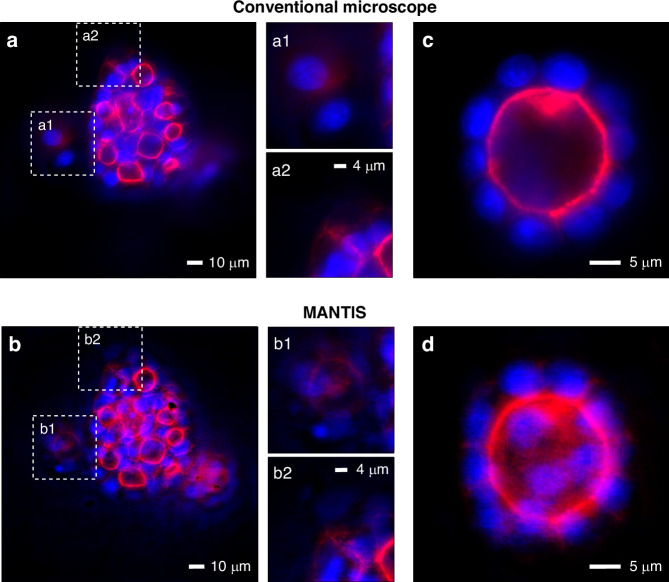
**MDCK II spheroids imaged with a conventional wide-field microscope and MANTIS. a** Conventional wide-field fluorescence image acquired as a single exposure without axial scanning from a ≈50 μm thick MDCK II spheroid embedded in Matrigel, stained for F-actin (ATTO 643, magenta) and nuclei (DAPI, blue). (a1, a2) Zoomed views of the dashed regions in (a), showing limited native DoF and out of focus background. **b** Snapshot MANTIS reconstruction of the same FoV. (b1, b2) Corresponding zooms, showing improved contrast and sharper cellular features. **c** Additional wide-field single exposure example highlighting a ring-like actin cortex. **d** Corresponding MANTIS reconstruction, preserving cell boundaries and nuclear structure across the spheroid thickness. Scale bars: 10 μm in (**a**, **b**); 4 μm in (a1, a2, b1, b2); 5 μm in (**c**, **d**)

To further assess the DoF performance of MANTIS, we imaged 3D-cultured MDCK II cells (≈50 μm thick), which form spheroids with hollow lumens rather than a monolayer. The samples were labeled with ATTO 643-phalloidin (F-actin) and DAPI (nuclei). Figure 5 compares conventional wide-field images and MANTIS reconstructions for two representative fields of view. In the conventional microscopy images (Fig. 5a, c), only a narrow axial region of each spheroid lies within the native DoF, so out-of-plane structures appear blurred and low contrast. The actin cortex is diffused, and neighboring cells tend to merge, and in Fig. 5c, the ring-like cortex around the lumen is only partly in focus, and several nuclei are dimmed or smeared. With MANTIS (Fig. 5b, d), we obtain that the spheroids remain sharper. This is most evident in the zoomed regions marked by the white dashed boxes (Fig. 5a1, a2 vs. Fig. 5b1, b2): MANTIS preserves cell boundaries and clearly resolves individual DAPI-labeled nuclei throughout the 3D structure, whereas the conventional images show smeared cortices and merged cell profiles. As an additional reference, we acquired conventional wide-field axial z stacks and performed Richardson-Lucy deconvolution, reporting maximum intensity projections of the deconvolved volumes for visualization (Supplementary Section 10).

Furthermore, to evaluate performance on a specimen type not used during training and to assess the generalizability of MANTIS, we imaged a 16 *μ*m mouse kidney section (FluoCells Prepared Slide 3, F24630) stained with three fluorescent labels (nuclei with DAPI, cell membranes with WGA conjugated Alexa Fluor 488, F-actin with Alexa Fluor 568). The cellular length scales are comparable to those in MDCK epithelial samples, but kidney tissue shows a more heterogeneous organization, with renal tubules and glomerular regions. DAPI labels nuclei, AF488 delineates renal tubules, and AF568 highlights F-actin, providing a three-channel, multispectral test in tissue. For each FoV, we acquired two datasets: a conventional wide-field image and a measurement using a microscope equipped with the learned multispectral meta-optic. Only the meta-optic measurements were processed with the end-to-end trained D-CNN; the wide-field images are shown without post-processing and serve as the baseline. Both systems imaged the same tissue regions at identical out-of-focus planes, revealing that the meta-optic system, combined with the CNN, restores the fine tubular and cytoskeletal structure that remains unresolved in conventional wide-field microscopy.

**Fig. 6:**
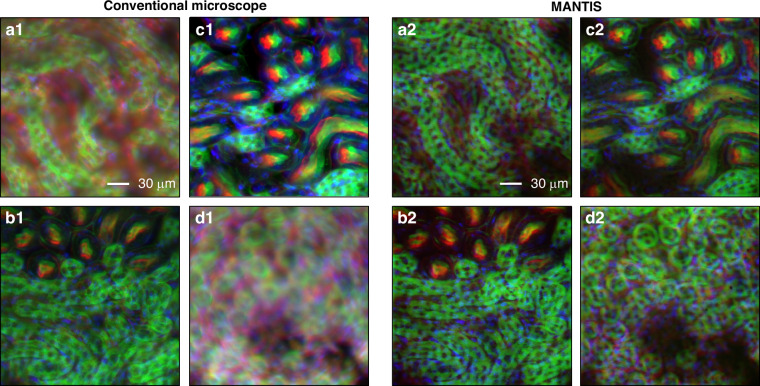
**Imaging a 16** **μm thick mouse kidney section**. Each pair of panels (**a1**-**a2**, **b1**-**b2**, **c1**-**c2**, **d1**-**d2**) shows co-registered wide-field and MANTIS images acquired at the same stage position, while the four pairs correspond to different FoV within the tissue section. Conventional wide-field images (**a1**–**d1**) show depth-dependent blur and mixed in-plane and out-of-plane contributions, with some planes appearing locally sharp while other structures remain defocused due to the limited native DoF relative to the section thickness. MANTIS reconstructions (**a2**–**d2**) improve contrast and preserve fine structural features more consistently across the field and depth range, enabling EDoF imaging without serial refocusing. RGB maps AF568 → R, AF488 → G, and DAPI → B. Scale bars, 30 μm

Figure 6 compares conventional wide-field imaging with MANTIS at matched axial offsets. For each pair, the wide-field image and the corresponding MANTIS measurement are acquired at the same stage position, with axial offsets defined relative to a common reference plane. We define *z* = 0 μm as the nominal mid-plane of the 16 μm-thick section. To establish this reference, we first located the coverslip facing surface by translating the stage toward the objective until the section surface came into focus, then moved 8 μm into the specimen and re-optimized the wide-field focus. This mid-plane focus position was used as *z* = 0 μm, and all reported *z*_*i*_ values are stage offsets relative to this plane. In Fig. 6, the pairs (a1, a2) to (d1, d2) were acquired at *z*_*i*_ = −7 μm, −3.5 μm, +3.5 μm, and +7 μm, respectively, and correspond to different FoV within the section.

Across these axial offsets, the wide-field images (a1–d1) exhibit increasing defocus blur and strong out-of-focus background. Even when some structures are near best focus, their visibility is reduced by fluorescence from the rest of the section. In contrast, the MANTIS reconstructions (a2 to d2) retain higher contrast and sharper boundaries over the same offsets, consistent with EDoF behavior, particularly in dense regions such as glomeruli and surrounding tubules.

Finally, we emphasize that MANTIS provides an EDoF reconstruction rather than optical sectioning and does not refocus to distinct depth planes within the tissue. The purpose of varying *z*_*i*_ in Fig. 6 is to evaluate robustness to axial displacement relative to the reference plane. The four examples correspond to different FoV, so differences in the high contrast structures reflect specimen variability and local optical heterogeneity across the section.

## Discussion

We introduce MANTIS, a computational fluorescence microscopy system that jointly learns a multispectral meta-optic and a D-CNN through end-to-end training. In the optical path, a TiO_2_ nanopillar meta-optic is placed at the conjugate Fourier plane of a 4f relay, where it applies wavelength-dependent pupil phase coding to reshape the system PSF across depth and wavelength. The recorded sensor image is then processed by a modified UNet to reconstruct the object. In a conventional high NA microscope, defocus introduces a second-order wavefront error that imposes a depth-dependent quadratic phase at the pupil, causing the PSF to vary rapidly with axial position. In parallel, chromatic dispersion produces wavelength-dependent focal shifts and aberration changes, so the optimal focus plane and blur characteristics differ across spectral channels. In MANTIS, the learned phase profile compensates these effects by shaping the pupil such that the modulation transfer function (MTF) varies more slowly with defocus over the target range. This preserves informative measurements under strong defocus across wavelengths and improves the conditioning of the inverse problem. The full system is trained using a physics-based differentiable forward model and a multi-term loss, enabling optical encoding and computational decoding to co-optimize for depth robust and spectrally consistent reconstructions.

MANTIS delivers EDoF performance over targeted ranges of 25–75 μm in simulation, and we experimentally validate the 50 μm configuration. Across depth and wavelength, PSNR varies only modestly with axial position and cross-spectral changes are minimal, while SSIM trends track the structural content of each channel. Measured PSFs confirm reduced axial sensitivity and strong chromatic stability relative to a conventional microscope. Reconstructions of biological specimens further demonstrate that fine cellular structure remains visible and sharp throughout the extended focal range.

We expect MANTIS to generalize to new specimens if the microscope configuration is fixed and the fluorophore emission falls within the same four spectral bands used during training. Consistent with this, we imaged a mouse kidney cryosection, an unseen tissue type with different morphology from the MDCK data and obtained robust reconstructions. Generalization may degrade when imaging conditions deviate substantially from training, for example, in highly scattering or strongly autofluorescent specimens, under markedly different spatial statistics, or when emission lies outside the trained bands. For new specimen types within similar emission bands, fine-tuning the reconstruction network with a calibration dataset acquired through the meta-optic can recover performance. By contrast, changes in fluorophore labeling or the spectral configuration alter the emission spectra and thereby change the wavelength-dependent PSFs encoded by the meta-optic. In this case, end-to-end retraining may be required to match the new spectral regime.

While our results quantify performance under high NA and large defocus coefficients, it is important to recognize that prior EDoF methods are often optimized for different points on the tradeoff between DoF range, lateral resolution, and spectral bandwidth. For example, some approaches prioritize near diffraction-limited and depth uniform PSFs over a shorter EDoF, whereas others target a larger EDoF but reduced lateral resolution, and many are optimized for a single wavelength. In contrast, MANTIS is designed to operate under substantially stronger defocus while jointly satisfying multispectral constraints. This choice inherently trades off peak lateral sharpness at a single plane for improved reconstruction stability across depth and wavelength. We evaluate this primarily using image-level performance, reporting PSNR and SSIM versus depth, together with the corresponding wide-field baseline curves under the same reference definition. Additionally, we report the FWHM measured from 1 *μ*m beads as an indicator of the raw optical PSF and its depth dependence. Because MANTIS includes computational reconstruction, this bead-based FWHM does not directly represent the final lateral resolution after postprocessing.

To assess the benefit of multispectral learning, we also evaluated a single-wavelength model trained at 601 nm with a one-channel reconstruction network. In simulations, this configuration provides higher spatial fidelity near its design wavelength, but experimentally, the reconstructions show strong wavelength dependence, with image quality varying across channels. In contrast, the multispectral model learns jointly across channels and maintains uniform performance, enabling robust multispectral fluorescence imaging.

In conclusion, MANTIS combines end-to-end multispectral co-optimization of the meta-optic and reconstruction network with an MTF-guided frequency-domain initialization and a well-defined Fourier-plane sampling strategy. Unlike earlier EDoF approaches that trade off either NA or sample complexity, MANTIS enables high NA fluorescence imaging of thick, multi-label biological specimens in a single shot, providing a practical route to high-resolution multispectral EDoF imaging. Future work will incorporate measured system aberrations into the design, extend the framework to broader spectral coverage and larger EDoF ranges, and advance the system toward three-dimensional reconstruction of volumetric samples.

## Methods

### Microscope model

The microscope model simulates the forward image formation process by receiving high-resolution fluorescence image patches *GT*_*λ*_ as input, where each patch is a fixed-size spatial crop extracted from a full FoV image, together with a depth *z*, and generating the corresponding sensor image *SI*_*z*,*λ*_. At each iteration, a depth and wavelength-dependent *PSF*_*z*,*λ*_ is computed based on wave optics theory. The sensor image is then formed by convolving the ground-truth image with the simulated PSF:

5 $$S{I}_{z,\lambda }=G{T}_{\lambda }* PS{F}_{z,\lambda }+\eta$$

where *η* denotes sensor noise. A detailed derivation of the forward imaging model and PSF formulation is provided in Section 5 in the Supplementary.

To compute each PSF, the model constructs a spatially varying phase profile *ϕ*_*z*,*λ*_, which consists of a defocus phase term ${\phi }_{z,\lambda }^{DF}$ and a wavelength-dependent phase modulation term ${\phi }_{\lambda }^{M}$. The modulation term ${\phi }_{\lambda }^{M}$ is determined by the underlying geometry of the meta-optic, which is learned during training, and is computed using a differentiable spectral lookup function implemented in the meta-optics simulator part, as illustrated in Fig. 1b:

6 $${\phi }_{\lambda }^{M}={\rm{Interp}}({U}_{\gamma },{U}_{{\phi }_{\lambda }},\gamma )$$

where *γ* denotes the meta-optic radius vector, *U*_*γ*_ is the set of discrete geometric widths, and ${U}_{{\phi }_{\lambda }}$ contains the corresponding wavelength-dependent phase delays obtained from electromagnetic simulations of meta-optic unit cells. Details of the unit cell library construction and the corresponding electromagnetic simulation results are provided in Section 6 in the Supplementary.

Before end-to-end training, we pre-optimize the meta-optic alone for 30 epochs using an EDoF frequency-domain objective. From the simulated stack *PSF*_*z*,*λ*_, we compute the MTF at each depth *z* and wavelength *λ*, normalize by the DC term, and form a DC-normalized radial profile over normalized spatial frequency *ρ* ∈ [0, 1], denoted *M*_*λ*_(*ρ*, *z*). We compare this profile to a target passband *T*(*ρ*) that is unity up to a cutoff *ρ*_*cut*_. For each wavelength, depth errors are aggregated with a log-sum-exp operator as

7 $${{\mathcal{L}}}_{{\rm{EDOF}},\lambda }=\frac{1}{\beta }\log \mathop{\sum }\limits_{z}\exp \left(\alpha {\left\Vert {M}_{\lambda }(\rho ,z)-T(\rho )\right\Vert }_{1}\right)$$

Here *β* > 0 controls the aggregation across depths: A large *β* approaches *max*_*z*_ (most defocused plane dominates), whereas a small *β* approaches the average over depths. In practice, we use a large *β* so that “soft-max” pooling emphasizes the worst-case depth and drives the design toward meta-optic parameters that maintain image quality at the largest defocus. The result is a single depth-robust score ${{\mathcal{L}}}_{{\rm{EDOF}},\lambda }$ for each wavelength. To balance performance across wavelengths, we then aggregate the per-channel scores as follows:

8 $${{\mathcal{L}}}_{{\rm{EDOF}}}={\lambda }_{focus}{L}_{EDOF,{\lambda }^{* }}+\left(1-{\lambda }_{focus}\right)\frac{1}{\eta }\log \mathop{\sum }\limits_{\lambda }\exp \left(\eta ,{L}_{EDOF,\lambda }\right)$$

where λ^*^ = *argmax*(*L*_*EDOF*,*λ*_) is the channel that currently limits and *η* > 0 controlling the aggregation across channels (large *η* approaches the maximum over *λ*, small *η* approaches the mean). The coefficient *λ*_*focus*_ ∈ [0, 1] places extra weight on the limiting channel. This pre-optimization yields a multispectral EDoF seed that produces sensor images with manageable blur and stabilizes the subsequent end-to-end training. We also tested a commonly used cubic-phase initialization^25^, which produced extremely blurred sensor images under the large defocus and reduced the effective signal for the learned D-CNN. In contrast, a flat *pi*-phase initialization, combined with the EDoF-MTF pre-optimization, provides a physically meaningful starting point that evenly distributes contrast across depth and wavelength. By comparison, for a conventional wide-field microscope equipped only with a learned post-processing network (and no meta-optic), the situation is analogous but more constrained: native defocus blur dominates the measurement, leaving the sensor data severely out of focus, and the inverse problem poorly conditioned.

During training, the reconstruction loss is back-propagated through the microscope model, enabling gradient-based optimization of the meta-optic design. Specifically, loss gradients with respect to the PSF, $\partial {\mathcal{L}}/\partial PS{F}_{z,\lambda }$, are propagated through the wave optics model to compute the derivative with respect to the nanopillar geometry, $\partial {\mathcal{L}}/\partial \gamma$. In this parameterization, an effective edge width parameterizes the meta-optic radius vector *γ*:

9 $$\gamma ={\gamma }_{{\rm{init}}}+\Delta \gamma$$

where *γ*_init_ is a fixed initialization based on the frequency-domain EDoF objective, and Δ*γ* is a learnable perturbation. The full 2-D radius map is formed by azimuthally replicating radius vector *K* times,

10 $${\Gamma }_{full}=Ro{t}_{K}(\gamma )$$

where *Rot*_*K*_( ⋅ ) is a fixed linear replication operator with no learnable parameters. A differentiable edge-to-phase lookup, $Interp({U}_{w},{U}_{{\phi }_{\lambda }},\gamma )$, then yields the per-wavelength phase *ϕ*_*λ*_. By the chain rule, $\partial {\mathcal{L}}/\partial \gamma$ is obtained from $\partial {\mathcal{L}}/\partial PS{F}_{z,\lambda }$ through the wave-optics model and the lookup, updating the learned radius vector and producing an end-to-end optimized meta-optics. Detailed gradient derivations are provided in Section 7 in the Supplementary.

### D-CNN

The proposed deblurring module takes the sensor image *SI*_*z*,*λ*_ and predicts a four-channel reconstructed image aligned to the high-resolution ground-truth fluorescence images *GT*_*λ*_. We adopt a three-level encoder-decoder UNet with skip connections^33^, inspired by recent advances in biomedical image restoration^17,29–31^. Before the encoder, each spectral channel is normalized using InstanceNorm to decouple features from channel-specific intensity and contrast variations that naturally occur in multispectral data due to different fluorophores or sensor responses. The channels are then linearly mixed by a 1 × 1 convolution initialized to identity. This 1 × 1 convolution provides the network with a flexible, learnable starting point for spectral feature extraction, allowing it to automatically discover the optimal linear combination of the normalized channels before spatial processing begins. A Fourier-domain channel-attention block then computes per-channel gates from the magnitude spectra of the feature channels and uses them to reweight the features, providing wavelength-aware balancing at minimal cost.

Each encoder block consists of a 2 × 2 max-pooling operation followed by two 3 × 3 convolutional layers, each with batch normalization and ReLU activation. The feature depth increases progressively from 32 to 128 across the encoder. For the decoder, bilinear upsampling is applied at each stage, followed by the same double-convolution structure to recover spatial resolution while reducing feature depth. A final 1 × 1 convolution is used to project the output to four channels, corresponding to the spectral components. The network learns a residual image through a hyperbolic tangent (tanh) activation function, which is added to the input *SI*_*z*,*λ*_ to reconstruct the deblurred output. The deblurred output is passed through a Clamp layer to keep the pixel values between 0 and 1. This step helps avoid unrealistic results and maintains the training stability.

To ensure memory efficiency, the network operates on 512 × 512 pixel patches corresponding to specific depth and wavelength combinations. Each input patch is initially convolved with the corresponding PSF and subsequently cropped to 512 × 512 pixels to suppress boundary artifacts introduced by the convolution operation.

### End-to-end learning

The end-to-end network is trained on fluorescence images of MDCK epithelial cells (Fig. 2) acquired with a Nikon ECLIPSE Ti2 inverted microscope and a CFI Plan Apo 60 × /1.4 NA oil-immersion objective (see Section “Sample preparation” for full preparation and staining details). To introduce spectral variability and prevent overfitting, we expanded the dataset with natural images from the Hyperspectral Image Dataset^34^. The final mixed dataset contains 423 MDCK fluorescence images and 37 hyperspectral natural images, corresponding to approximately 92 percent and 8 percent of the dataset, respectively. The hyperspectral images were converted to four-channel inputs by selecting bands whose center wavelengths are closest to our four fluorescence emission channels. In total, the mixed dataset comprises 460 images of size 512 × 512 pixels, split into training, validation, and test sets at 80%, 10%, and 10%. This mixed dataset encourages the network to learn spatial and spectral features across varying scales, thereby improving its robustness and generalization. During training, data augmentation is performed through random rotations, flips, and brightness variations. At each iteration of the training and validation stages, a randomly selected in-focus ground-truth image patch is assigned to one of *K* discrete depth positions *z*_*i*_ ∈ (*z*_1_, . . . , *z*_*K*_), uniformly distributed in diopters across the target DoF range. The assigned depth z selects the corresponding *PSF*_*z*,*λ*_ used to synthesize *SI*_*z*,*λ*_. The ground-truth is depth independent, that is *GT*_*z*,*λ*_ = *GT*_*λ*_ for all *z*. During each forward pass, output images are computed for each selected depth and wavelength, and the loss is accumulated and averaged across all combinations.

We use a fixed multi-term loss to capture fidelity, structure, edges, and coarse content. For each (*z*, *λ*) patch:

11 $$\begin{array}{rcl}{\mathcal{L}} & = & \frac{1}{K\,C}\mathop{\sum }\limits_{z,\lambda }\left[{w}_{\mathrm{RMSE}}\,\mathrm{RMSE}\,\left(O{I}_{z,\lambda },G{T}_{z,\lambda }\right)+{w}_{\mathrm{SSIM}}\,\left(1-\mathrm{SSIM}\,\left(O{I}_{z,\lambda },G{T}_{z,\lambda }\right)\right)\right.\\ & & \,+\left.{w}_{\mathrm{grad}}\,{\ell }_{\mathrm{grad}}\,\left(O{I}_{z,\lambda },G{T}_{z,\lambda }\right)+{w}_{\mathrm{LP}}\,\mathrm{MSE}\,\left({\phi }_{\mathrm{LP},k}(O{I}_{z,\lambda }),{\phi }_{\mathrm{LP},k}(G{T}_{z,\lambda })\right)\right]\end{array}$$

where *K* and *C* are the numbers of depth planes and spectral channels. *ℓ*_*grad*_ matches horizontal and vertical finite differences to preserve edges, and *ϕ*_*LP*,*k*_ denotes a fixed low-pass filtered representation used in an MSE term to align large-scale structure and intensity balance. During learning, RMSE enforces pixel level fidelity and provides the dominant optimization signal under our weighting, while SSIM, gradient matching, and low-pass MSE act as complementary regularizers.

We optimize the end-to-end network using two Adam optimizers^35^: one for the optical parameters (no weight decay) and one for the D-CNN parameters with *L*2 regularization. The total training objective is:

12 $${\mathcal{J}}={\mathcal{L}}+\beta {\left\Vert {\theta }_{{\rm{deblur}}}\right\Vert }_{2}^{2}$$

while the optical parameters are not regularized.

Since the input undergoes varying levels of blur across depth and wavelength, the network naturally adapts the meta-optic to produce PSFs that are less sensitive to defocus and spectral changes. As a result, no additional loss term is needed to enforce PSF similarity within the targeted depth range explicitly.

### Sample preparation

We evaluated three specimen types. (i) MDCK II epithelial cells cultured on *#*1.5H coverslips and immunolabeled for lamin A/C (Alexa Fluor 488) and vimentin (Alexa Fluor 568); F-actin (Atto 640) was labeled with phalloidin and nuclei with DAPI. (ii) A fixed mouse kidney cryosection (16 μm; FluoCells Prepared Slide *#*3, *F*24630) labeled with DAPI, Alexa Fluor 488, and Alexa Fluor 568. (iii) Polyacrylamide hydrogels (11 *kPa*) with embedded 1 μm fluorescent beads used to measure the PSF, assess lateral resolution (FWHM). Full protocols, reagent sources, catalog numbers, dilutions, and timings are provided below.

Madin-Darby canine kidney II (MDCK II) epithelial cells were maintained in media consisting of Minimum Essential Medium (MEM) with GlutaMAX and Earle’s salts (#41090028, *Gibco, USA*), 10 % FBS (#10500064, *Gibco*) and 83 U/ml penisillin/streptomycin (#15140122, *Gibco*) at 37 ^∘^C in 5% CO_2_. The cells were passaged using Trypsin-EDTA (0.25 %, #25200056, Gibco) and plated at approximately 6 × 10^4^ cells/cm^2^ on 22 × 22 mm glass coverslips (No. 1.5H, Marienfeld, Germany) in 6-well plates (#3506, *Corning, USA*) and cultured for 5 days. All the following steps were conducted at room temperature. Cells were fixed in 4% paraformaldehyde (v/v, #157–8, Electron Microscopy Sciences, USA) in 1x PBS for 10 min and washed 3 times in PBS for 5 min. Permeabilization of the cells was done in 0.5% Triton-X-100 (v/v, *#T*8787, Sigma-Aldrich, USA) in 0.5% BSA in PBS for 10 min, followed by blocking in 3% bovine serum albumin (BSA) (w/v, *#P*06-139210, PAN-Biotech, Germany) in PBS for 1 h. Primary antibodies lamin A+C [131C3] (#*ab8984*, abcam, UK) in 1:300 dilution and vimentin (*#ab*92547, abcam) in 1:200 dilution in 3% (w/v) BSA in PBS were incubated for 1 h. Samples were washed three times for 10 min, first in the permeabilization buffer, then in PBS, and finally again in the permeabilization buffer. Secondary antibodies goat anti-mouse Alexa Fluor 488 (#*A*-11029, Invitrogen, USA) and goat anti-rabbit Alexa Fluor 568 (#A11011, Invitrogen, USA) in 1:200 dilution in 3% BSA (w/v) in PBS were incubated for 1 h in dark. Cellular actin was labeled with ATTO 643 Phalloidin (#*AD643*-81, ATTO-TEC, Germany) in 1:100 dilution with the secondary antibodies. The samples were washed 2 times in PBS for 10 min and stained for DAPI in 1:1000 dilution in ddH_2_O. Finally, the samples were washed twice in PBS and mounted in Prolong diamond antifade mountant (*#P*36961, Invitrogen). Samples were kept overnight in room temperature and then stored at 4 ^∘^C. Samples were imaged on a Nikon ECLIPSE Ti2 with a CFI Plan Apo 60 × /1.4$, NA oil-immersion objective.

In addition, MDCK II cells were cultured in a 3D environment, where cells form spheroids with empty lumens instead of a monolayer. Cells were cultured and passaged as described above. Matrigel (*#*354277, Corning) was thawed overnight on ice and kept below 4 ^∘^C prior to gelation. Cells were mixed with Matrigel, and 40 μl droplets were pipetted into the wells of a chambered coverslip suitable for microscopy (*#*80807, Ibidi), containing approximately 8000 cells in each gel. The gels were polymerized for 15 min inside the cell culture incubator. 300*μ*l of cell culture media was added to each well, and the samples were cultured for 7 days, with media changes every 2–3 days. Cells were fixed in 4% paraformaldehyde with 0.01% Glutaraldehyde (#340855, *Sigma-Aldrich*) in 1× PBS solution pre-warmed to 37 ^∘^C for 20 min at 37 ^∘^C. The following steps were conducted at room temperature. The samples were washed 3 times in PBS for 5 min. Cells were permeabilized for 45 min and labeled with ATTO 643 Phalloidin and DAPI, as described above. The samples were stored at 4 ^∘^C in 1x PBS.

A commercially prepared slide was used (*FluoCells Prepared Slide* #3; F24630, Thermo Fisher Scientific/Invitrogen). The slide contains a 16 μm cryostat section of mouse kidney labeled with DAPI, WGA conjugated to Alexa Fluor 488, and Phalloin conjugated to Alexa Fluor 568. Slides were imaged as supplied, following the vendor’s instructions; no additional processing was performed.

Polyacrylamide (PA) hydrogels with Young’s modulus of *E* = 11 kPa were prepared following the protocol of^36^. The hydrogels were prepared between two glass coverslips. Both the bottom coverslips (22 × 22 mm, No. 1.5H, *#*0107052, Marienfeld) and top coverslips (*d* = 13 mm, #631–0150, VWR, USA) were washed with 2% HelmaneX III (*#Z*805939, Hellma, Germany) in ddH_2_O solution for 30 min in sonicator (CPX3800H-E Bransonic, Branson, USA), rinsed with dH_2_O and EtOH, and dried with compressed nitrogen. The bottom coverslips were treated with0.3% v/v bind-silane (3-(trimethoxysilyl) propyl methacrylate (*#M*6514, Sigma-Aldrich) and 5% v/v glacial acetic acid solution in 95% ethanol to facilitate PA gel adhesion. Next, 10% acrylamide (stock 40%, #1610140, Bio-Rad Laboratories, USA) and 1% Bis-acrylamide (Bis) (stock 2%, *#*1610142, Bio-Rad Laboratories) were mixed with PBS and degassed in a vacuum. 0.04% v/v of fluorescent beads (1 *μ*m tetraspecks, *#T*7282, Invitrogen) was added to the solution. To initiate PA polymerization, 0.2% v/v of TEMED (*#*1610800, Bio-Rad Laboratories) and 1% v/v of ammonium persulfate (APS) stock solution in ddH20 (10% w/v, *#A*3678, Sigma-Aldrich) were mixed to the solution. Twenty six microliters droplet was pipetted on the bottom coverslip and the top coverslip was placed on top of the droplet to construct approximately *h* = 200 μm gels. The PA gels were polymerized for 15 min in room temperature and after polymerization were stored in PBS at 4 ^∘^C.

### Meta-optics fabrication

The learned multispectral meta-optic design is fabricated using electron beam lithography and reactive ion etching on a fused silica substrate coated with a high-refractive-index TiO_2_ layer. Details of the fabrication steps are provided in in Section 8 in Supplementary. As a reference, the end-to-end learned single-wavelength meta-optic design was fabricated by the same process. Both meta-optics are integrated into the microscope at the Fourier plane of a 4*f* system via a relay configuration; a detailed schematic of the experimental setup is provided in Supplementary Fig. 1. This placement ensures that phase modulation occurs in the angular domain, enabling PSF shaping across both spatial and spectral dimensions.

## Supplementary information

Supplementary for Extended-Depth Multispectral Fluorescence Microscopy with Co-Designed Meta-optics and Reconstruction

## Data Availability

The MDCK fluorescence dataset acquired in this work has been deposited in Zenodo: https://zenodo.org/records/18609221. Third-party data from the Hyperspectral Image Dataset are publicly available as cited in the manuscript. Additional data supporting the findings of this study are available from the corresponding authors upon reasonable request.

## Code availability

Training and inference code for the reconstruction network, together with pretrained model weights and example configuration files, are available on GitHub: https://github.com/humeyracaglayan/MANTIS. A black box version of the optical forward model, which maps ground-truth images to simulated sensor measurements, is also provided. The differentiable implementation of the optics forward model is available from the authors upon reasonable request, subject to intellectual property constraints.
