# Supplementary material for: Multispectral extended depth-of-field fluorescence microscopy with co-designed meta-optics and neural reconstruction
Source: Light Sci Appl. 2026 May 19;15:242. doi: 10.1038/s41377-026-02337-y (PMC13187408; doi:10.1038/s41377-026-02337-y)
Supplement: Supplementary file 1 — Supplementary for Extended-Depth Multispectral Fluorescence Microscopy with Co-Designed Meta-optics and Reconstruction [file 41377_2026_2337_MOESM1_ESM.docx]

**Supplementary Information for
Multispectral Extended Depth-of-Field Fluorescence Microscopy with Co-designed Meta-Optics and Neural Reconstruction**

Ipek Anil Atalay Appak^1,2^, Haobijam Johnson Singh^1^, Sanna Korpela^3^, Teemu O. Ihalainen^3^, Erdem Sahin^4^, Christine Guillemot^2^, and Humeyra Caglayan^1,5,*^

^1^ Physics Unit, Tampere University, Faculty of Engineering and Natural Sciences, Tampere, 33720, Finland.

^2^ INRIA Rennes, Bretagne Atlantique Research Centre, Rennes, 35042, France.

^3^ Bio Unit, Faculty of Medicine and Health Technology, Tampere University, Arvo Ylpön katu 34, Tampere, 33520, Finland.

^4^ Signal Processing Research Centre, Tampere University, Tampere, 33720, Finland.

^5^ Photonic Integration Group, Department of Electrical Engineering, Eindhoven University of Technology, Eindhoven, 5600 MB, The Netherlands.

^*^ Corresponding author’s Email: [h.caglayan@tue.nl](mailto:h.caglayan@tue.nl)

#### Table-of-contents

[Section 1 | Experimental Setup 3](#_Toc222930762)

[Section 2 | Single-wavelength learning 4](#_Toc222930763)

[Section 3 | Lateral FWHM calculation from the measured PSFs 6](#_Toc222930764)

[Section 4 | Ablation Study 7](#_Toc222930765)

[Section 5 | Derivation of the forward imaging model and PSF formulation 8](#_Toc222930766)

[Section 6 | Meta-optics unit cell library and electromagnetic simulation 9](#_Toc222930767)

[Section 7 | Gradient derivation for meta-optics learning 10](#_Toc222930768)

[Section 8 | Meta-optics fabrication 11](#_Toc222930769)

[Section 9 | Oversampling 11](#_Toc222930770)

[Section 10 | Widefield z stack deconvolution and maximum intensity projection 11](#_Toc222930771)

[References 12](#_Toc222930772)

1. Experimental Setup

Our widefield microscope setup is based on sequential multicolor epi-fluorescence imaging technique in inverted configuration. The set-up consists of four single colour LEDs (M405L4, M470L5, M565L3, M625L4, Thorlabs) as illumination sources which are configured with three additional external achromatic lenses (L1, L2, AC254-050-AB-ML; L3, AC254-0100-AB-ML, Thorlabs) in the light path to achieve Kohler illumination.

The LEDs are first collimated using aspheric lenses (ACL2520U-A, Thorlabs) and then directed towards the external lens arrangement using appropriate dichroic mirrors (DM1- FF435-Di01-25x36, DM2- FF518-Di01-25x36, DM3- FF593-Di03-25x36; Semrock). The motorised excitation filter-wheel (FW103H/M, Thorlabs) selects the appropriate excitation filter (FF01-392/23-25, FF01-474/27-25, FF01-554/23-25, FF01-635/18-25, Semrock) and the transmitted colour is then directed upwards towards the objective (LUMFLN60XW, Evident) through the multiband dichroic filter (FF409/493/573/652-Di02-25x36, Semrock). The objective, mounted on a piezo stage (PFM450E, Thorlabs) excites the specimen. The fluorescent signal is collected by the same objective which is then captured by the imaging optics to form an image of the specimen. The emission filter (FF01-440/40-25, FF01-514/30-25, FF01-595/31-25, FF01-698/70-25, Semrock, all house in the motorised emission filter-wheel, FW103H/M, Thorlabs) along with the multiband dichroic filter ensures the incident colour is fully blocked and only the fluorescent signal is collected by the tubelens (TTL180-A, Thorlabs) which then focuses the light at the camera (ORCA-Fusion C14440-20UP, Hamamatsu) sensor plane to form the image. The imaging side is divided into two channels through the beamsplitter (BSW10R, Thorlabs) where in one channel, an additional 4f relay system (AC254-075-A-ML, Thorlabs) is included and the metasurface is mounted at the Fourier plane. This split optical path on the imaging side provides simultaneous capture of standard microscope image and the metasurface modulated image. The whole set-up is automated for sequential multichannel XYZ scan imaging using a single user interface (UI) software controlled.


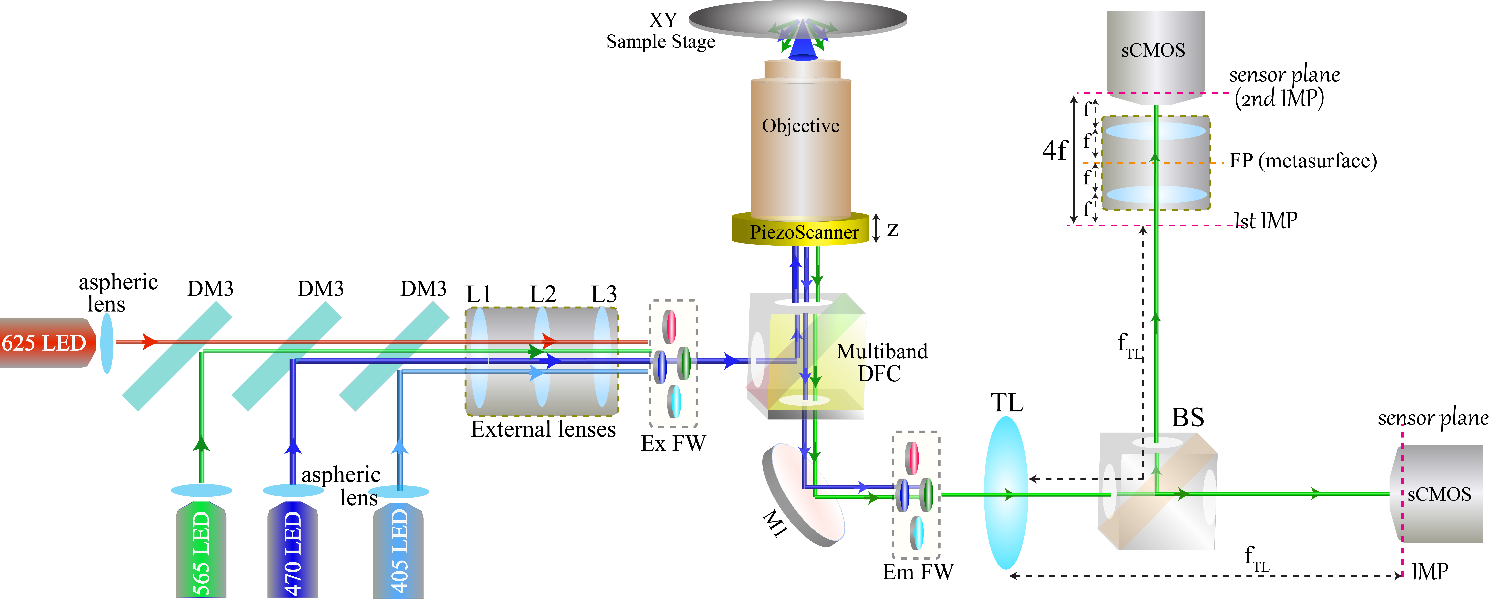


Fig. S1. Schematic of our imaging setup. DM-dichroic mirror; L1, L2, L3-achromatic lenses; Ex/Em FW- excitation/emission filter-wheel; DFC-multicolour dichroic filter cube; M1-mirror; TL-tubelens; BS-beam splitter; FP-fourier plane; IMP-intermediate image plane; objective-60x, 1.1NA, water immersion.

1. Single-wavelength learning

We trained a single wavelength model for 601 nm over a 50 µm DoF using our earlier UNet implementation. It achieves higher resolution at its design wavelength due to the lower task complexity, but exhibits wavelength dependence, as shown in Fig. S2.

We performed repeated PSF measurements for the fabricated single-wavelength meta-optic using the same illumination and experimental conditions. Fig. S3 shows well-formed PSFs across the tested wavelengths, reflecting stable alignment and robust device performance.

To complete the comparison, we experimentally tested the learned single wavelength meta-optic and reconstructed the captures with a one channel CNN. The measured PSFs are consistent across wavelengths, yet the reconstructions show strong chromatic dependence. Although training used 601 nm, the highest experimental PSNR and SSIM were observed at 681 nm. Because the PSFs are comparable, this gain is unlikely to come from optical sharpness. The more likely causes are channel specific signal-to-noise and scene statistics. The 681 nm channel delivered higher effective signal with lower background. In addition, the structures labeled in the 681 nm band are higher contrast and spatially smoother, which aligns better with the priors learned by the UNet. These factors explain the better reconstruction at 681 nm despite training at 601 nm. See Fig. S4.


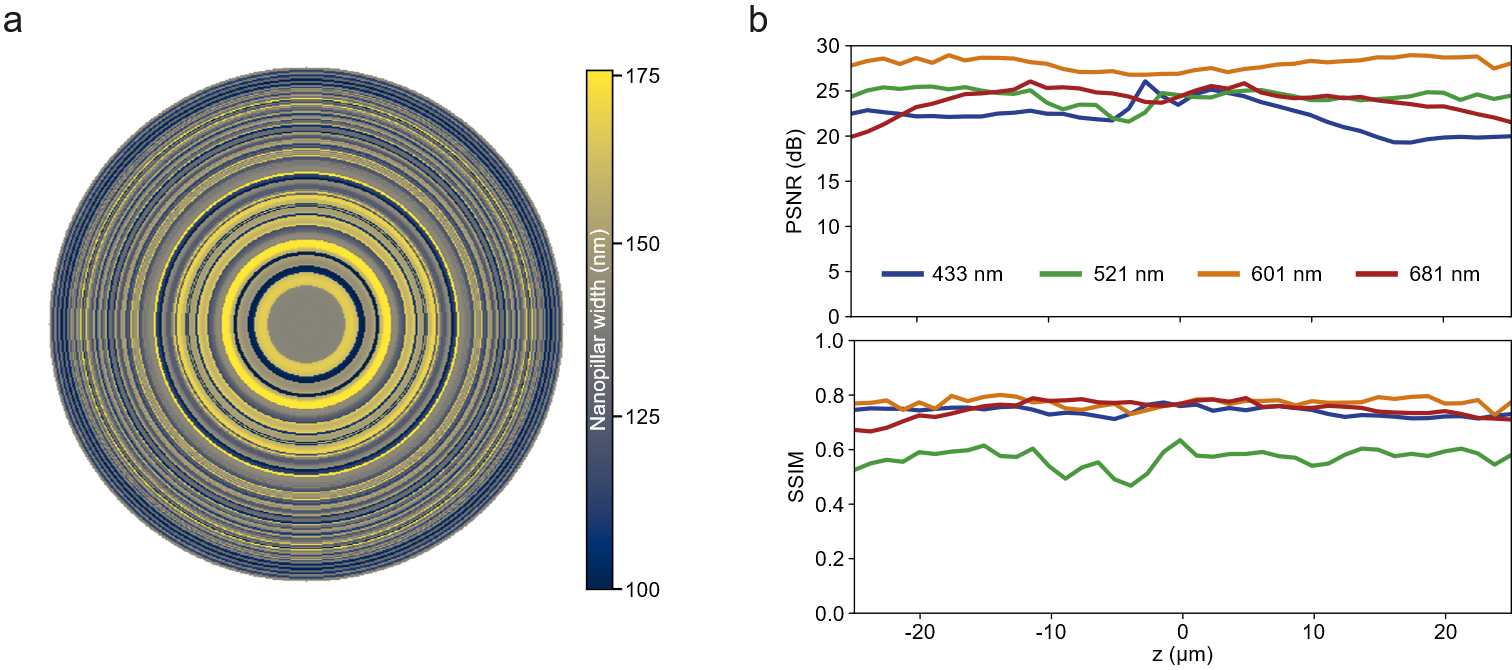


Fig. S2. Single-wavelength simulation results. Single-wavelength simulation results. (a) End-to-end learned multispectral meta-optic layout visualized as a nanopillar-width map. (c) PSNR and SSIM versus axial depth for the single-wavelength design, evaluated across 41 planes within the target DOF and multiple wavelengths.


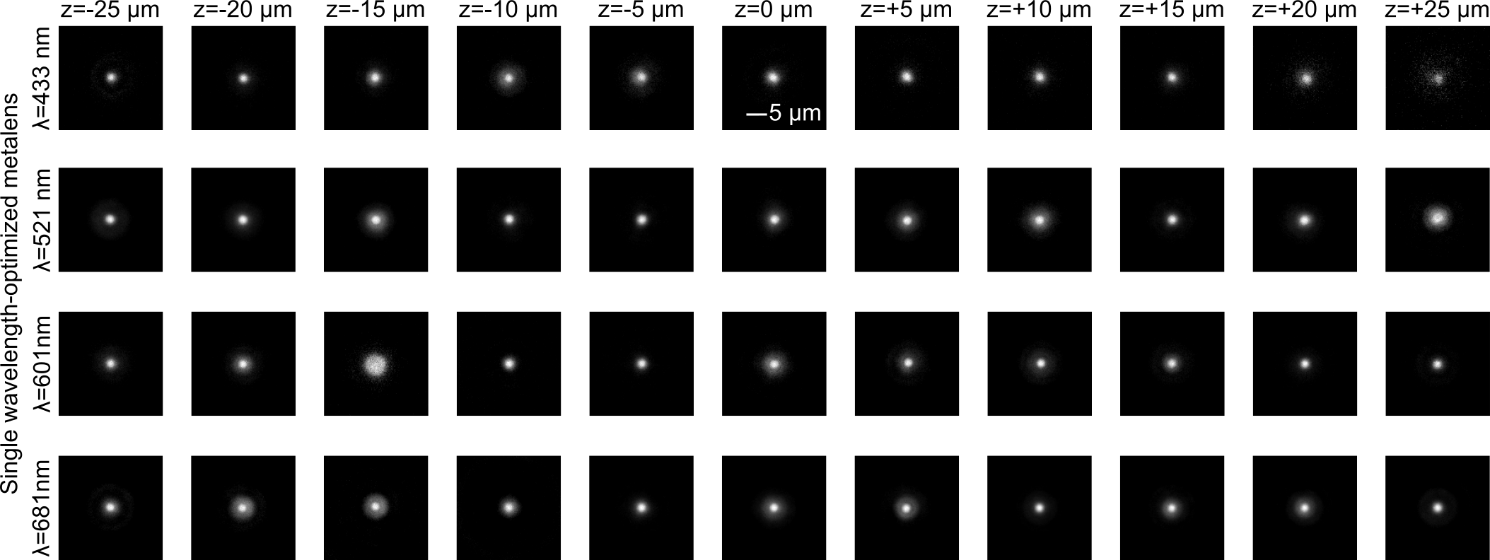


Fig. S3. Experimental point spread functions (PSFs) for the fabricated single-wavelength (601 nm) meta-optic across the 50 µm target DOF. Rows correspond to illumination wavelengths and columns to 11 axial positions from -25 µm to +25 µm relative to best focus. PSFs were measured from 1 µm fluorescent beads and are shown with identical intensity scaling.


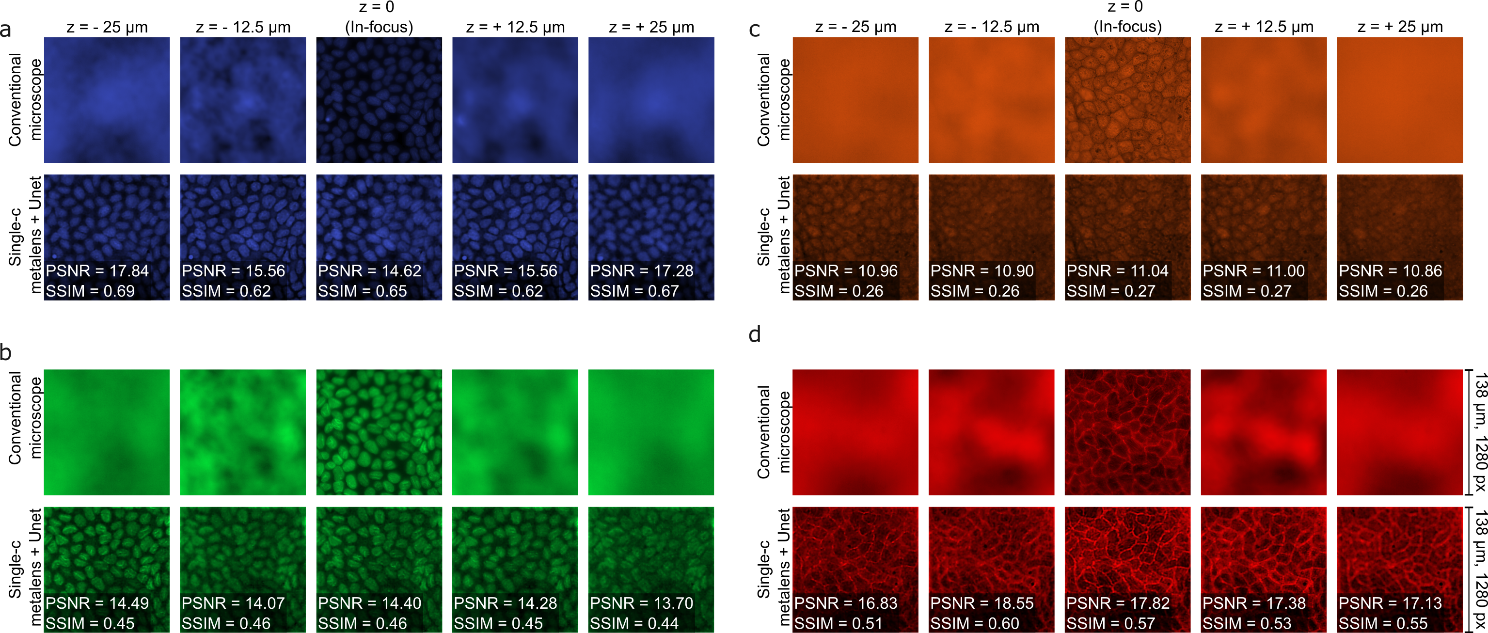


Fig. S4. Single-wavelength design (601 nm), experimental MDCK cell imaging. Each subpanel shows the conventional wide-field microscope measurement (top) and the 1-channel CNN reconstruction with learned metalens (bottom) for five representative depth planes within the target DOF.

1. Lateral FWHM calculation from the measured PSFs

We estimate the FWHM of $1 \mu m$ beads by fitting a 2D Gaussian to each measured PSF at every wavelength and depth. For each bead image, we locate the brightest pixel and crop a fixed-size ROI around it. We then fit an axis-aligned elliptical Gaussian

$$G\left( x,y \right)=A\exp\left( -\frac{\left( x-x_{0} \right)^{2}}{2\sigma_{x}^{2}}-\frac{\left( y-y_{0} \right)^{2}}{2\sigma_{y}^{2}} \right)+c$$

using least squares, and convert pixel units to micrometers with the calibrated camera pitch. For a 1D Gaussian, $\text{FWHM}=2\sqrt{2\ln2}\sigma$. For each wavelength and depth, we show *x,y* line profiles through the PSF peak with the fitted curves and annotate the FWHM (Fig. S5). The learned multispectral meta-optic maintains comparatively stable FWHM with defocus and across wavelengths, consistent with simulations (Fig. S6).

Quantitatively, over the evaluated depth range, the lateral FWHM is 1.42 ± 0.38 µm (blue), 1.73 ± 0.70 µm (green), 1.91 ± 0.95 µm (orange), and 1.94 ± 1.08 µm (red), where ± denotes the standard deviation over depth. The corresponding maximum absolute deviations from the best focus plane are 1.46, 2.60, 2.99, and 3.90 µm, respectively. To quantify chromatic variation, at each depth we compute the standard deviation of FWHM across the four wavelengths. This cross wavelength spread has a mean of 0.57 µm and a maximum of 1.68 µm over the full depth range.


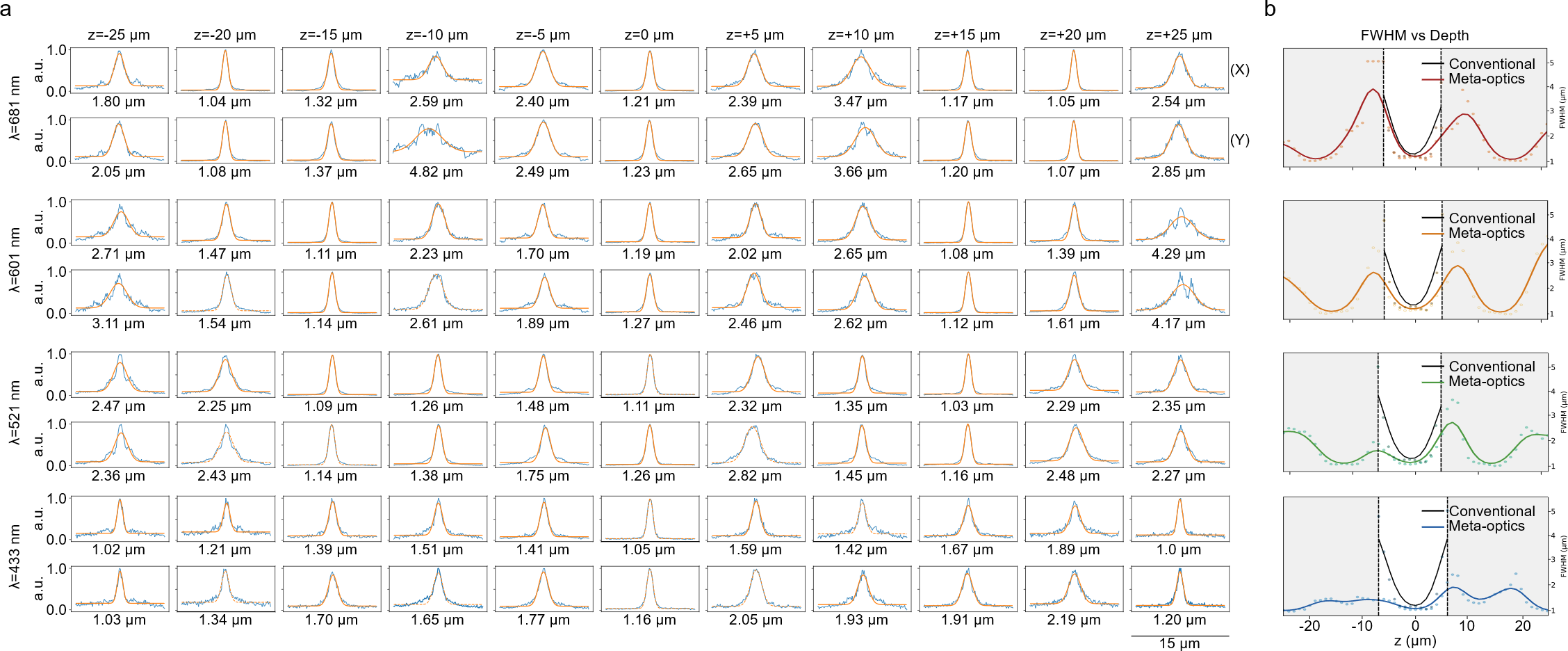


Fig. S5. Multispectral metalens FWHM calculation for measured PSFs. (a) For each depth and wavelength, x and y line profiles through the PSF peak are shown with the corresponding Gaussian fits, and the resulting FWHM values are annotated. (b) Measured lateral FWHM as a function of depth, evaluated at 1 µm axial sampling. FWHM is reported as the average of the *x* and *y* FWHM values obtained by Gaussian fitting of the corresponding line profiles. Colored curves correspond to the meta-optic arm, and the black curve to the conventional widefield reference arm, computed using the same procedure. Grey shading marks depths where the widefield PSF becomes substantially broadened, with FWHM exceeding 5 µm.


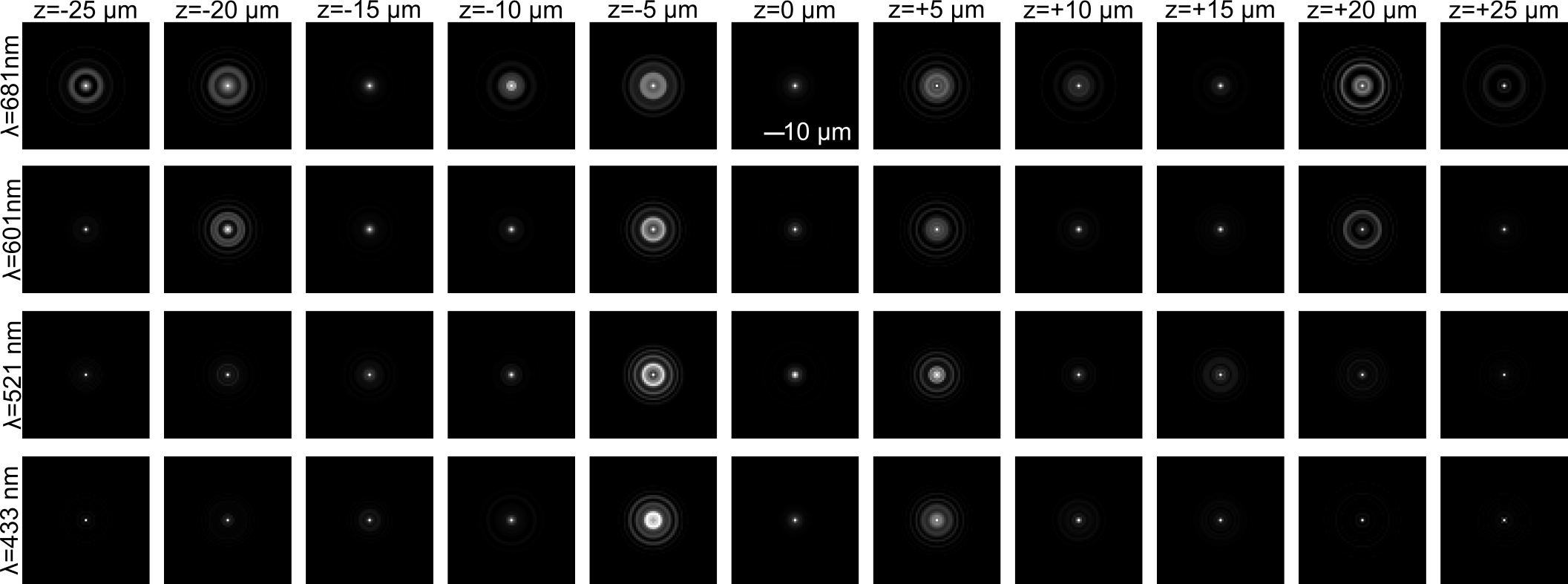


Fig. S6. Simulated PSFs of the learned multispectral meta-optic. Each row corresponds to a wavelength channel and each column to one of 11 axial planes spanning the target DoF. Intensities are shown with identical scaling. The set visualizes the depth behavior of the multispectral design and its wavelength-dependent ring structure while maintaining compactness across the DOF.

1. Ablation Study

The aim of this ablation is to isolate the roles of optical coding and computational decoding in a high NA, large defocus regime. Fig. S7a compares three experimental configurations at 521 nm across depth. In the first configuration (top row), where the meta-optic is removed and only the D-CNN operates on measurements from the conventional microscope, reconstructions are heavily blurred and exhibit poor spectral fidelity, indicating that the defocus at high NA produces measurements that the network cannot reliably invert. In the second configuration, where the D-CNN is omitted and only the learned meta-optic is used, the sensor images are modestly sharper than in the D-CNN only case yet still show substantial blur and loss of fine structure. The third configuration, corresponding to the full co-designed system MANTIS, yields clearly improved contrast and structural detail across all channels, indicating that robust multispectral EDoF performance relies on the meta-optics and the D-CNN acting together. Fig. S7b provides additional control using a purely conventional wide-field model. The top row shows the wide-field reference arm measurements, at the same axial positions, illustrating how image quality degrades with defocus in the baseline microscope. The bottom row shows reconstructions from a D-CNN that is trained and tested using a conventional wide-field forward model. In this setting, defocus blur increases rapidly with axial offset and the network fails to recover structure across the target range, leading to strongly smooth, low detail outputs away from focus. Together, these controls support that optical coding is required to keep the inverse problem sufficiently well-conditioned over the extended depth range.


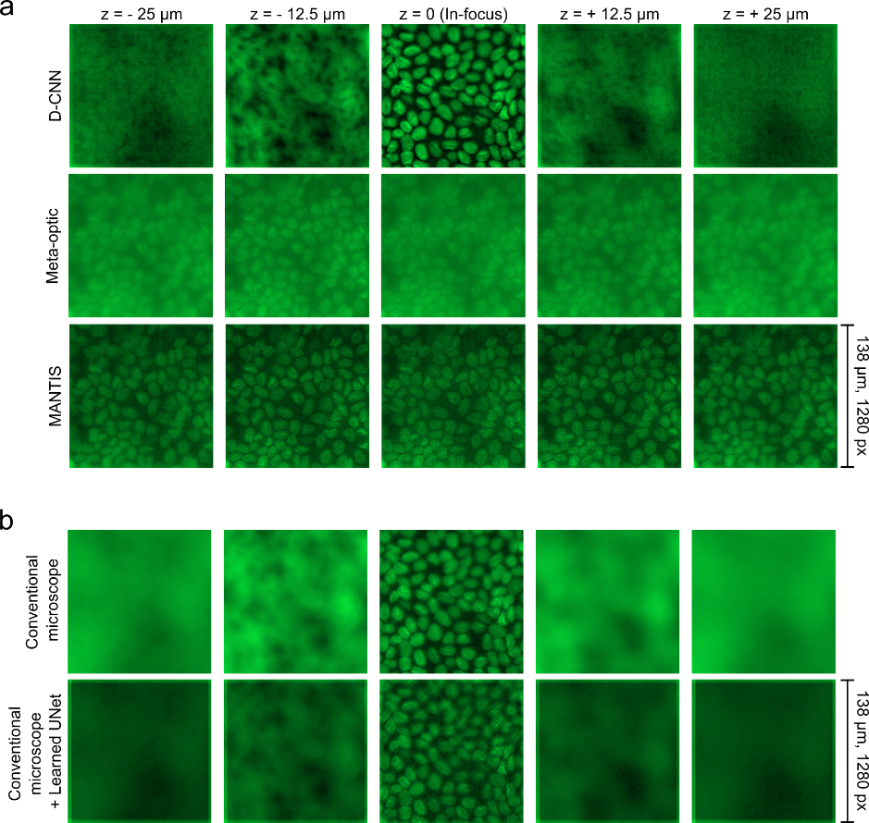


Fig. S7. Ablation study. Experimental comparison for MDCK cells at the 521 nm emission channel. Columns show axial positions *z* =- 25 ,-12.5,0,+12.5,+25 μm spanning a 50 μm depth range. Field of view 138 μm image size 1280 pixels. (a) Ablation to isolate the contributions of optical coding and computational decoding. Top row: widefield measurements from the reference arm (no meta-optic) processed with the D-CNN trained jointly with the meta-optic. Because the input does not match the forward imaging model seen during training, reconstructions degrade away from focus. Middle row: meta-optic arm measurements shown without reconstruction (coded sensor images). Bottom row: full MANTIS (meta-optic plus D-CNN), maintaining cellular contrast and nuclear structure across depth. (b) D-CNN only control using conventional widefield microscopy. Top row: Conventional widefield measurements from the reference arm. Bottom row: D-CNN trained and tested using a conventional widefield forward model (failure case). Under native high NA defocus, reconstructions collapse and fine structure is not recovered across depth, confirming that optical coding is required for reliable reconstructions.

1. Derivation of the forward imaging model and PSF formulation

## Original Microscope System (First 4f)

The field at the intermediate image plane $\left( x_{2},y_{2} \right)$ is computed from the pupil plane $\left( x_{1},y_{1} \right)$:

$$E_{1}\left( x_{2},y_{2};z,\lambda\right)\mathcal{=F}\left\{ A\left( x_{1},y_{1} \right) exp\left( i \phi_{\text{defocus}}\left( x_{1},y_{1};z,\lambda\right) \right) \right\}$$

where:

- $A\left( x_{1},y_{1} \right)$ is the aperture mask at the first Fourier plane (i.e., the tubelens entrance pupil), where the pupil diameter is given by $D_{\mathrm{pupil}}=2f_{1}\mathrm{NA}$.
- $\phi_{\text{defocus}}$ is the depth-dependent phase
- $\mathcal{F}\{\cdot\}$ is the 2D Fourier Transform.

## Second 4f System with Metalens

The second 4f system applies a phase modulation in the spatial frequency domain. Although the metalens is defined as a real-space mask $M\left( x,y \right),$ its physical placement in the Fourier plane of the second 4f system means it acts as a modulator of the frequency content of the image field:

1. Fourier transform to reach the second 4f Fourier plane:

$$E_{2}\left( x_{3},y_{3} \right)\mathcal{=F}\left\{ E_{1}\left( x_{2},y_{2} \right) \right\}$$

2. Multiply by metalens mask:

$$E_{3}\left( x_{3},y_{3} \right)=E_{2}\left( x_{3},y_{3} \right)\cdot M\left( x_{3},y_{3} \right)$$

where:

$$M\left( x_{3},y_{3} \right)=A_{\text{meta}}\left( x_{3},y_{3} \right)\cdot\exp\left( i\phi_{\text{meta}}\left( x_{3},y_{3};\lambda\right) \right)$$

1. Inverse Fourier transform to reach the sensor plane:

$$E_{4}\left( x_{4},y_{4} \right)=\mathcal{F}^{-1}\left\{ E_{3}\left( x_{3},y_{3} \right) \right\}$$

2. Final PSF:

$$\text{PSF}_{\text{final}}\left( x_{4},y_{4};z,\lambda\right)=\left| E_{4}\left( x_{4},y_{4} \right) \right|^{2}$$

## Full Expression

Combining all steps:

$$\text{PSF}_{\text{final}}\left( x_{4},y_{4};z,\lambda\right)=\left| \mathcal{F}^{-1}\left\{ M\left( x_{3},y_{3} \right)\mathcal{\cdot F}\left\{ \mathcal{F}\left\{ A\left( x_{1},y_{1} \right)\cdot\exp\left( i\phi_{\text{defocus}}\left( x_{1},y_{1};z,\lambda\right) \right) \right\} \right\} \right\} \right|^{2}$$

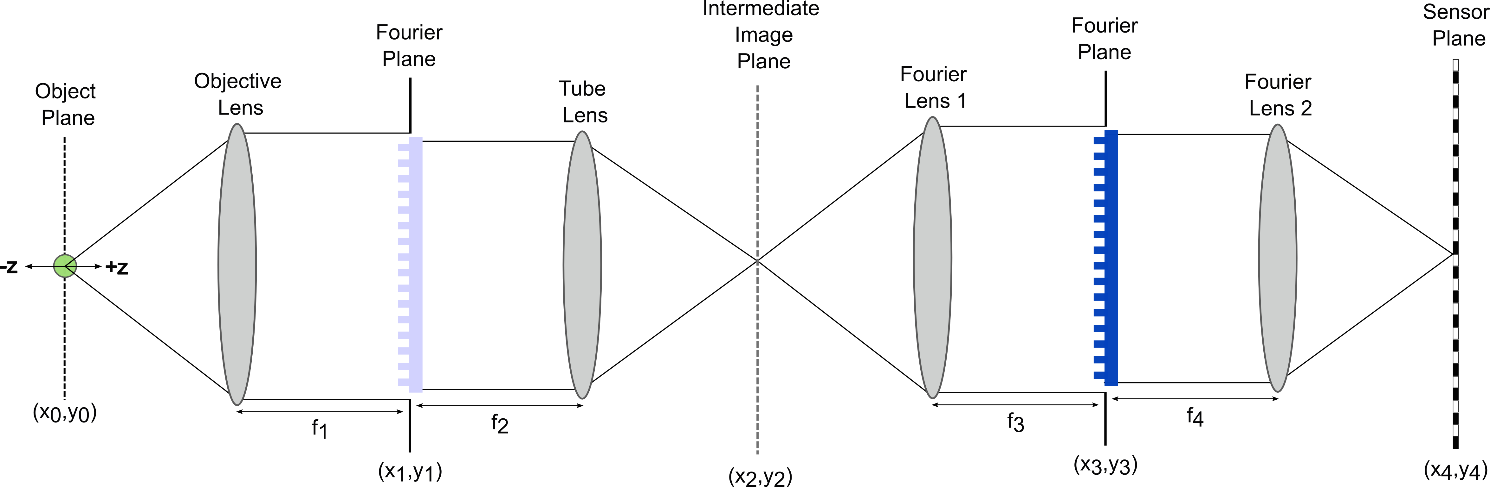


Fig. S8. EDOF microscope model schematic. An objective ${(f}_{1})$ and tube lens ${(f}_{2})$ form an intermediate image, which is relayed by a second $4f$ stage ${(f}_{3},f_{4})$ to the sensor. Meta-optics (dark blue) is placed at the second Fourier plane. Coordinate frames ${(x}_{0},y_{0})$ to ${(x}_{4},y_{4})$ mark each plane; $+z, -z$ denote object defocus.

1. Meta-optics unit cell library and electromagnetic simulation

For the meta-optics unitcell we use the propagation phase. A high index $\mathrm{Ti}O_{2}$ square pillar on glass acts as a dielectric waveguide and imparts a controllable phase delay to the transmitted field. We choose the pillar height ($h$) so that sweeping the edge width $U_{w}$ gives nearly $0$ to $2\pi$ phase coverage for each design wavelength. The $U_{w}$ range is $\left( 100 \text{nm}\leq U_{w}<P-50 \right).$ We keep the upper bound below the $P$ due to fabrication limits. We then optimize $h$ across all design wavelengths for higher transmission. Finite difference time domain simulations provide the complex transmission by sweeping $w$ at the chosen $h$. The unit cell curves in Fig. S8 show nearly full $2\pi$ phase with high transmittance over the band.


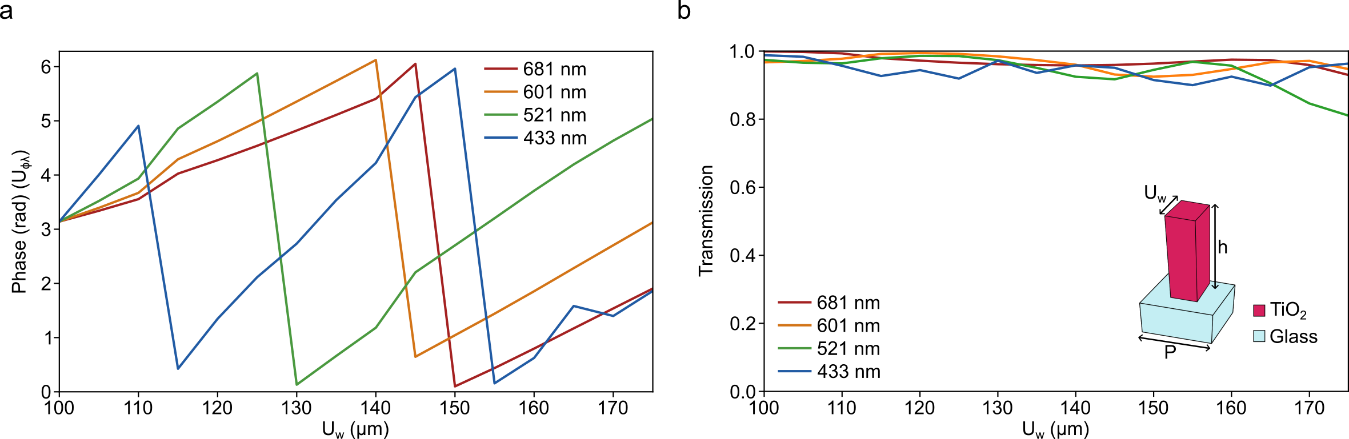


Fig. S9. Simulated unit cell lookup for a $\mathrm{Ti}O_{2}$ square pillar on glass. (a) Phase $U_{\phi_{\lambda}}$ versus edge width $U_{w}$ for the design wavelengths, showing near 0 to $2\pi$ coverage as $U_{w}$ varies from 100 to 175 nm. (b) Transmission versus $U_{w}$ for the same wavelengths with high transmittance over most of the range. Inset shows the scatterer geometry with width $U_{w},$ height $h=1000 nm,$ and period $P=225 nm.$

1. Gradient derivation for meta-optics learning

Starting from reconstruction loss, we train optics and D-CNN simultaneously. The reconstruction loss back-propagates through the wave-optics model:

$$\frac{\partial\mathcal{L}}{\partial\theta}=\frac{\partial\mathcal{L}}{\partial\text{PSF}z,\lambda}\cdot\frac{\partial\text{PSF}z,\lambda}{\partial\phi_{\lambda}}\cdot\frac{\partial\phi_{\lambda}}{\partial\gamma}\cdot\frac{\partial\gamma}{\partial\theta}$$

where $\phi_{\lambda}$ is the phase from the differentiable edge–phase lookup function. The differentiable edge–phase lookup function is defined as

$$\phi_{\lambda}\left( \gamma_{i} \right)=\left\{ \begin{aligned} U_{\left\{ \phi_{\lambda} \right\}}[1], &\gamma_{i}\leq U_{w}\left[ 1 \right] \\ \left( 1 - t \right)U_{\left\{ \phi_{\lambda} \right\}}[k]+ tU_{\left\{ \phi_{\lambda} \right\}}[k+1], &\gamma_{i} \in[U_{w}[k], U_{w}\left[ k+1 \right)) \\ \begin{matrix} U_{\left\{ \phi_{\lambda} \right\}}[K], & \gamma_{i}\geq U_{w}[K] \end{matrix} \end{aligned} \right.$$

$$t =\frac{\gamma_{i}-U_{w}[k]}{U_{w}[k+1]-U_{w}[k]}, k \text{ such that }U_{w}\left[ k \right]\leq\gamma_{i}< U_{w}\left[ k+1 \right]$$

where $U_{w}=\{\gamma_{k}{\}}_{k=1}^{K}$ is the sorted radius values, $U_{\phi_{\lambda}}={\Phi_{\lambda,k}}_{k=1}^{K}$ are the corresponding phase values, obtained from the meta-optics unit-cell library and $\gamma\in\mathbb{R}^{N}$denotes the meta-optic radius vector; $\gamma_{i}$ is its $i$*-th* element. Using this $\frac{\partial\phi_{\lambda}}{\partial\gamma}$ becomes

$$\begin{aligned} \frac{d\phi_{\lambda}}{d\gamma}= \left\{ \begin{aligned} 0, &\gamma_{i}\leq U_{w\text{ }}\left[ 1 \right]\text{ or }\gamma\geq U_{w}[K] \\ \frac{U_{\left\{ \phi_{\lambda} \right\}}[k+1]-U_{\left\{ \phi_{\lambda} \right\}}\left[ k \right]}{U_{w}[k+1]-U_{w}[k]},&\gamma_{i}\in\left( U_{w}\left[ k \right], U_{w}\left[ k+1 \right] \right) \end{aligned} \right. \end{aligned}$$

Using this parameterization, we learn with image domain losses for both the optical model and the D-CNN.

1. Meta-optics fabrication

The fabrication flow for the meta-optics is shown in Fig. S9. A $1000 nm$ $\mathrm{Ti}O_{2}$ film was first deposited onto a 400 µm thick glass substrate (Prazisions Glas & Optik GmbH) by ion-beam sputtering (Cutting Edge Coatings Navigator 700). A 250 nm thick PMMA 950K A4 electron-beam resist was then spin-coated and soft-baked at 180 °C for 90s. To suppress charging and to aid stage alignment during electron-beam lithography, a 20 nm Al conductive layer was deposited by e-beam evaporation (Instrumentti Mattila IM-9912). The metasurface pattern was written with a Vistec EPBG5000pES system. After exposure, the Al layer was stripped in AZ351B diluted with deionized water (1:5), and the resist was developed in a methyl isobutyl ketone:isopropanol solution (MIBK:IPA, 1:3). A 90 nm Cr hard mask was deposited by e-beam evaporation, and lift-off was performed overnight in Remover 1165 to define the mask. Pattern transfer into the $\mathrm{Ti}O_{2}$ was carried out by reactive-ion etching (Oxford Plasmalab System 100) using $SF_{6}$ and $\mathrm{Ar}$ flows of 7 and 13 sscm, respectively, at 100W RF power, 25 mTorr chamber pressure, and at $21 ^{\circ}C.$ Finally, the Cr mask was removed in a commercial chromium etchant (Sigma-Aldrich).


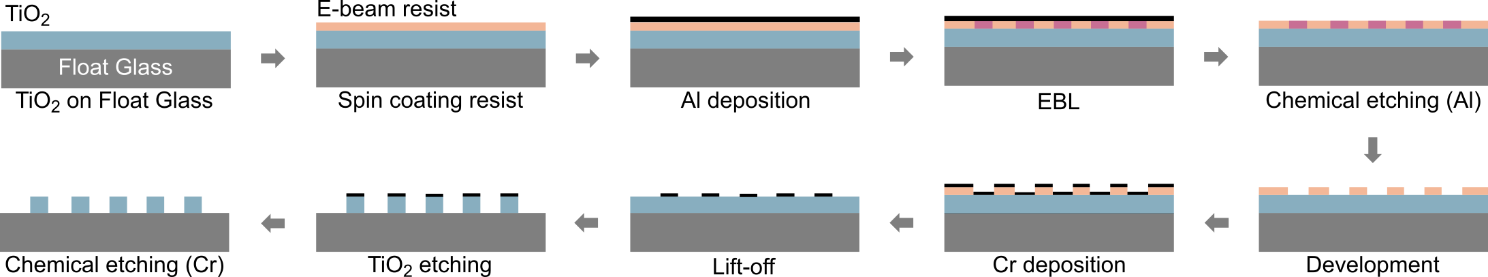


Fig. S10. Fabrication flow for the $\mathrm{Ti}O_{2}$ meta-optic on glass$.$

1. Oversampling

Supplementary Table 1 | Effect of Fourier-plane oversampling on reconstruction quality (50 µm DOF). All optical parameters are identical across runs, only the Fourier-plane sampling pitch $\Delta s$ changes. Reported PSNR/SSIM correspond to $\alpha=\{1,2,4,6\}; \alpha=4$ gives the best trade-off and maintains *a $\geq3\times$ SBP* margin.

| $\boldsymbol{\alpha}$ |  | **PSNR (dB)** | **SSIM** |
| --- | --- | --- | --- |
| 1 |  | 22.19 | 0.67 |
| 2 |  | 23.41 | 0.73 |
| 4 |  | 23.53 | 0.74 |
| 6 |  | 23.17 | 0.72 |

1. Widefield z stack deconvolution and maximum intensity projection

To obtain a volumetric reference for MDCK II spheroids, we reconstructed the 3D conventional widefield z stacks using Richardson Lucy deconvolution. Richardson Lucy is a widely used iterative deconvolution approach in fluorescence microscopy that uses a PSF based forward model to suppress out of focus blur and estimate the underlying object. Deconvolution was performed in Fiji using DeconvolutionLab2 [1], using experimentally measured widefield PSFs from our system as the forward model. DeconvolutionLab2 operates on a single channel, so each spectral channel was deconvolved separately. Unless otherwise stated, we used 50 iterations, selected empirically to balance deblurring and noise amplification, and stopped before full convergence. On our CPU workstation with 256 GB RAM, processing the volume in Fig. S11a required approximately 6 hours, whereas Fig. S11b required approximately 40 minutes for the two-channel stack. We report maximum intensity projections (MIP) of the deconvolved volumes for visualization.


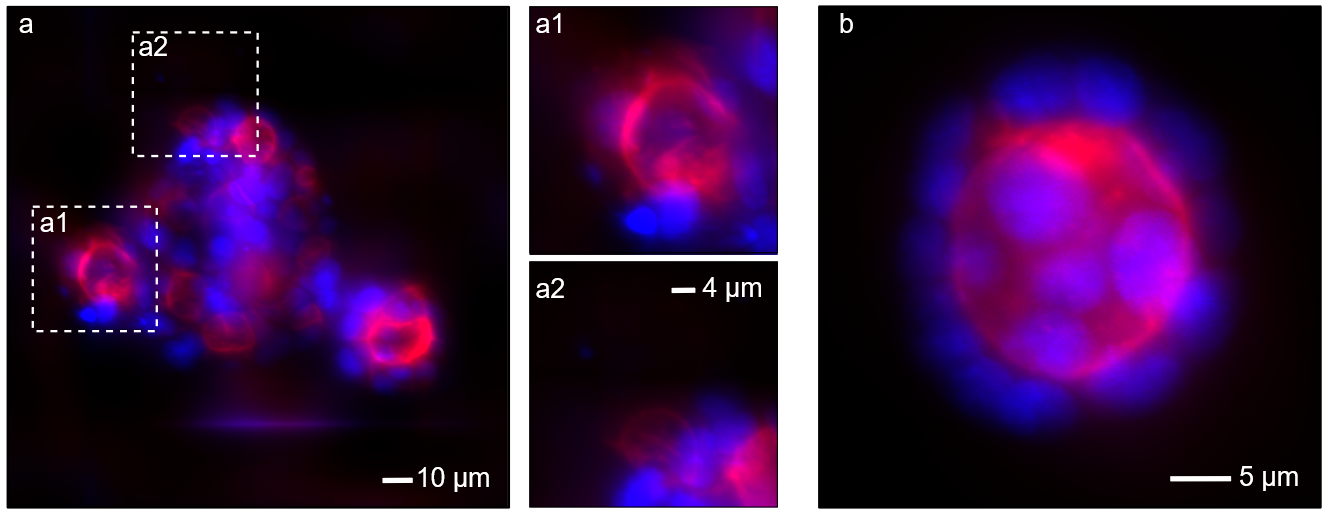


Fig. S11. Widefield z stack deconvolution for MDCK II spheroids. Conventional wide-field fluorescence z stacks of MDCK II spheroids were deblurred using Richardson Lucy deconvolution in Fiji with DeconvolutionLab2, using experimentally measured widefield PSFs from our system. MIP of the deconvolved volumes are shown for visualization. Scale bars as indicated in the panel.

References

[1] Sage, D. et al. DeconvolutionLab2: An open source software for deconvolution microscopy. Methods 115, 28 to 41 (2017).
